# Scaling Amphiphilicity with Janus Nanoparticles: A New Frontier in Nanomaterials and Interface Science

**DOI:** 10.3390/nano15141079

**Published:** 2025-07-11

**Authors:** Mirela Honciuc, Andrei Honciuc

**Affiliations:** “Petru Poni” Institute of Macromolecular Chemistry, 41A Grigore Ghica Voda Alley, 700487 Iasi, Romania; honciuc.mirela@icmpp.ro

**Keywords:** Janus nanoparticles, self-assembly, surface tension reduction, Pickering emulsions, solid-state foams

## Abstract

Janus nanoparticles (JNPs) extend the concept of amphiphilicity beyond classical molecular surfactants into the nanoscale. Amphiphilic behavior is defined by the presence of hydrophobic and hydrophilic moieties within a single molecular structure. Traditionally, such molecular structures are known as surfactants or amphiphiles and are capable of reducing interfacial tension, adsorbing spontaneously at interfaces, stabilizing emulsions and foams, and forming micelles, bilayers, or vesicles. Recent experimental, theoretical, and computational studies demonstrate that these behaviors are scalable to nanostructured colloids such as JNPs. Amphiphilic JNPs, defined by anisotropic surface chemistry on distinct hemispheres, display interfacial activity driven by directional wetting, variable interfacial immersion depth, and strong interfacial anchoring. They can stabilize liquid/liquid and liquid/gas interfaces, and enable templated or spontaneous self-assembly into supra-structures, such as monolayer sheets, vesicles, capsules, etc., both in bulk and at interfaces. Their behavior mimics the “soft” molecular amphiphiles but also includes additional particularities given by their “hard” structure, as well as contributions from capillary, van der Waals, hydrophobic, and shape-dependent forces. This review focuses on compiling the evidence supporting amphiphilicity as a scalable property, discussing how JNPs function as colloidal amphiphiles and how geometry, polarity contrast, interfacial interactions, and environmental parameters influence their behavior. By comparing surfactant behavior and JNP assembly, this work aims to clarify the transferable principles, the knowledge gap, as well as the emergent properties associated with amphiphilic Janus colloids.

## 1. Introduction

This is a short overview of the progress made in Janus nanoparticles (JNPs) research, specifically focusing on their interfacial properties as a manifestation of the scalable amphiphilic properties, and is mainly addressed to the scientists from the field of colloids, interfaces, and surfactants, as well as to the scientists and engineers from different fields who are interested in nanoscale, self-assembly phenomena and nanotechnologies.

**Figure 1:**
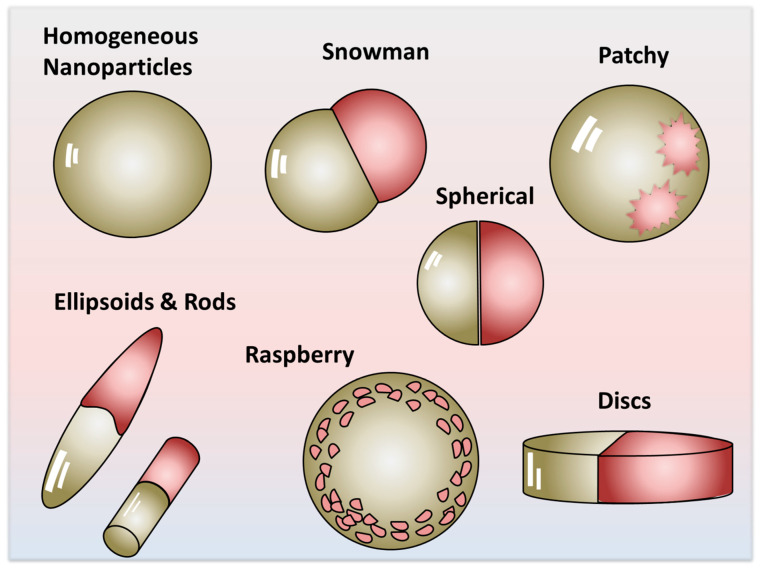
Representation of various architectures and morphologies that can be considered Janus particles.

Janus nanoparticles are a distinct subclass of engineered colloids characterized by an intrinsic structural and chemical asymmetry—comprising at least two regions or domains with differing physical or chemical properties. This anisotropy endows them with multifunctionality, as each of the constituting parts can be loaded with a bulk or surface property—e.g., electric conductivity, magnetism, polarity—which differ or contrast with one another, leading to tunable properties merely by changing the proportion, the volume, or the mass of the constituting parts [1,2]. This multifunctionality, tunability in properties, and versatility in applications is not accessible to isotropic or homogeneous nanoparticles (HNPs). Depending on the synthesis strategy and constituent materials, JNPs can exhibit a wide range of morphologies, including snowman-shaped [3], dumbbell-like, hybrid orbital analogs [4], mushroom structures [5], as well as rod- and disk-shaped forms [6]. Among these, the dumbbell and snowman geometries are most commonly associated with the canonical or strictly Janus morphology, owing to their clear biphasic separation and spatial orientation, as illustrated in Figure 1. In contrast to HNPs, which display uniform surface chemistry, JNPs offer a unique platform for designing anisotropic interactions and directional functionalities, leading to amphiphilic systems, self-propelled motors, nanoparticles with variable properties, etc.

One of the most immediate and intrinsic features of JNPs is their ability to display amphiphilic behavior when one domain is hydrophilic and the other hydrophobic. This structural duality imparts surfactant-like properties at the colloidal scale, enabling their use as solid amphiphiles capable of stabilizing liquid/liquid or liquid/gas interfaces. Accordingly, such Janus particles have been referred to as “solid-state amphiphiles,” expanding the classical concept of amphiphilicity beyond the molecular scale. Their dual nature allows them to selectively localize at immiscible interfaces, such as oil/water or water/air, where they can significantly lower interfacial tension and form stable Pickering emulsions or Pickering foams [7,8].

Amphiphilic JNPs play an enabling role in the fabrication of advanced hybrid materials. Those JNPs exhibiting spontaneous interfacial adsorption are particularly effective as stabilizers in Pickering emulsion polymerization systems. These systems facilitate the synthesis of nanostructured microparticles with tailored surface morphology, which have proven effective for applications such as heavy metal ion adsorption and removal from contaminated water [9]. Multifunctional JNPs loaded with contrasting bulk or surface properties have enabled the development of conductive polymer-based nanostructures [10,11] and multifunctional composites with sensing capabilities [12], as well as the opportunity to generate scalable asymmetric films with controlled surface topographies [12]. Compared to traditional surfactants, Janus particles offer greater structural stability, gradual adjustability of interfacial properties, and enhanced resistance to desorption under dynamic conditions [8], possibility to generate ultra-stable emulsions [13] and solid-state foams [14,15,16].

The binary and modular nature of JNPs allows for extensive customization of their surface chemistry and interfacial behavior by tuning the geometry and the chemistry of each lobe. By functionalizing each Janus hemisphere/lobe independently, one can design particles with asymmetric chemical reactivity, differential wettability, or spatially contrasting electronic and optical properties. This feature is particularly valuable for guiding directional self-assembly, enabling selective interactions, and constructing higher-order, supra-colloidal architectures [10,16]. JNPs have been shown to assemble into diverse morphologies—such as micelles, vesicles, and capsules—with dynamic, switchable structures responsive to environmental stimuli [16,17]. Their ability to mimic surfactant self-assembly, while offering size and surface modularity, makes them attractive components for stimuli-responsive materials and adaptive systems [18,19].

Moreover, the anisotropic structure of JNPs is suitable for applications in which active matter manifests directional motion in chemical or physical gradients. For instance, JNPs have been engineered into self-propelled nanomotors that utilize catalytic asymmetry to generate propulsion, with potential utility in targeted drug delivery, environmental remediation, and microscale robotics [20].

A further advantage of Janus nanoparticles lies in their compatibility with a broad range of surface modification strategies. The selective grafting of functional groups, ligands, or polymers onto one hemisphere enables the creation of particles with tailored surface energies, charge distributions, or specific biological functionalities [1,2,21]. This flexibility has been harnessed in numerous applications, including optoelectronics, biosensing, and biointerfaces. For example, JNPs combining semiconducting and insulating domains not only exhibit tunable dielectric properties suitable for integration into responsive composites and flexible electronic systems [1,2,21], but also asymmetric synergistic catalysis [22]. The latest research shows the growing interest in JNPs and their application in a plethora of different fields, from which we cite a few remarkable results, such as JNPs for superhydrophobic film formation on tooth surface to prevent bacterial attachment [23], biofilm eradicating JNPs for suppressing inflammation and controlling infection [24], JNPs for theranostics [25], JNPs as elastomer coatings for improving impact performance [26], amphiphilic JNPs for foam fractionation of fluorinated pollutants [27], JNPs with tunable refractive index [28], JNPs capable of interfacial self-assembly and film-forming ability in encapsulation of oils [29], JNPs with tunable amphiphilicity [30], JNPs as catalysts for reduction of CO_2_ into value-added chemicals [31], JNPs for in vitro treatment of tumors [32], JNPs for enhanced oil recovery (EOR) [33], etc.

While we have highlighted the advantages of amphiphilic JNPs and their potential for applications such as enhanced oil recovery [34], targeted drug delivery [35,36,37,38], theranostics [39], medical imaging [40], environmental remediation applications [31,41], and interfacial engineering [42], we also note that there are still challenges in their wide spread deployment. The synthesis of uniform, monodisperse JNPs with scalable yields is still limited in some cases by cost, complexity, and batch variability. In biomedical applications, concerns regarding cytotoxicity, biocompatibility, and persistence in the organism must be elucidated for many different platforms of JNPs. Studies show that even small differences in particle anisotropy, surface chemistry, or aggregation behavior can lead to drastically different biological outcomes [36]. Efforts are underway to not only address their limitations but also uncover and elucidate the JNPs’ ability to interact with its surroundings in a biological environment and aquatic media, as well as to prove their true utility.

To summarize, JNPs are an emerging class of anisotropic nanomaterials whose asymmetric structure enables multifunctionality, directional interactions, and ability to gradually adjust contrasting properties through varying the particle’s morphology. Amphiphilic JNPs carry on the manifestation of amphiphilicity beyond the molecular scale. In addition, their tunable composition and surface chemistry make them valuable across disciplines, and ongoing advances in synthesis and applications are broadening their impact on both fundamental science and applied technologies. Finally, it is the choice of material in JNPs that determines their suitability for specific applications. For example, metal–metal JNPs, such as Au—Pt or Au—Fe_3_O_4_, combine plasmonic and magnetic properties and are explored for use in theranostics, bio-imaging, and magnetic field-guided drug delivery systems. Ceramic–ceramic JNPs, including TiO_2_—Fe_3_O_4_, SiO_2_—ZnO, or other combinations, can exhibit photocatalytic activity and magnetic responsiveness, making them candidates for environmental remediation, water purification, and photocatalysis. Silica-based JNPs, particularly those asymmetrically functionalized with hydrophobic/hydrophilic or reactive groups, are amphiphilic and chemically stable and can be widely deployed in Pickering emulsions and foam stabilization. Polymer–polymer JNPs, such as those obtained via seeded emulsion polymerization, are particularly suited for emulsion and foam stabilization, as well as generating stimuli-responsive interfaces.

## 2. Short Historical Account—Development of JNPs

The concept of biphasic or asymmetric nanoparticles with unique properties—different from those expected from homogeneous nanoparticles, later termed ‘Janus’ particles after the two-faced Roman god—originated in the late 1980s to early 1990s. The scientific metaphor of an amphiphilic, Janus-like grain was first explicitly proposed by the Nobel Laureate Pierre-Gilles de Gennes in 1991 as a Janus particle exhibiting two distinct physical or chemical faces. However, the practical exploration of such anisotropic colloids predates this conceptual framing.

The first experimentally realized Janus particles were reported in 1985 by Lee et al. [43], who synthesized asymmetric polystyrene/poly(methyl methacrylate) (PS/PMMA) latex particles via a seeded emulsion polymerization process. Later, Chen et al. [44] investigated the reaction parameters and conditions leading to formation of PS/PMMA and polystyrene/poly(ethyl methacrylate) (PS/PEMA) JNPs via seeded emulsion polymerization and the role of interfaces [45]. These early efforts laid the groundwork for developing synthetic strategies to produce particles with dual functionality via the seeded emulsion polymerization method [46,47].

In 1988, Casagrande and Veyssi [48,49] prepared micron-sized ‘‘Janus beads” with one side hydrophilic and the other hydrophobic by selective surface modification, via masking technique, and investigated for the first time their interfacial behavior at the suggestion of the Pierre-Gilles de Gennes.

Then, an important reference point in the development of the field was the seminal work performed by Müller and colleagues in 2001, which developed a new and scalable method for the synthesis of Janus soft polymer nanoparticles via self-assembly of amphiphilic polymers [50,51]. Later, solid JNPs derived from cross-linking of polymer micelles [52] and their self-assemblies were investigated [53]. This synthesis technique later enabled the fabrication of spherical, cylindrical, or disk-like Janus nanoparticles [6], and for the first time, their interfacial activity could be investigated. Müller and coworkers were also the first to employ the JNPs in stabilization and polymerization of Pickering emulsions [54]. The seminal works emerged from this group provided a significant impulse to the development of this field [55,56].

In early 2010s, Lattuada and Hatton [57], as well as Walther and Mueller [58], were among the pioneers to publish review works in the emerging field and offered the first classification of Janus particle synthesis methods, which include self-assembly, masking, phase separation, microfluidics, etc. Each approach allowed control over composition, shape, and surface functionality. Because these methods were currently reviewed and evaluated in different recent works [11,46,47], they will not be comprehensively covered here. Next, the focus of this work will be on uncovering aspects of amphiphilicity manifestations at the nanoscale and beyond.

## 3. Definition of Amphiphilic Property and Its Manifestation

Amphiphilicity, the property of a molecule that possesses both hydrophilic and hydrophobic domains, has long been considered a fundamental characteristic of molecular surfactants and block copolymers. The typical manifestations of amphiphilicity is spontaneous adsorption at interfaces, which is thermodynamically driven by entropy to minimize the energy of the interface between immiscible systems [59]. This ability to spontaneously partition at interfaces leads to subsequent types of amphiphilic manifestations, such as the stabilization of emulsions, generation of foams, and self-assembly into supra-structures. The types of self-assembled supra-structures formed by amphiphiles include micelles, inverse micelles, vesicles, membranes, rafts, etc. These structures have captured the attention of scientists across various technological fields, with applications as delivery vehicles for drugs and active compounds, as nanoreactors in enhanced oil recovery and in the formulation of cosmetic products, etc. The cellular membranes of living organisms are such self-assembled constructs, whereas phospholipids self-assemble, giving rise to a bilayer construct capable of holding cellular receptors and ion channels, providing flexibility and ensuring transport of metabolites in and out of the cell while preserving its structural integrity [60,61].

Often neglected, but extremely important, is the topology of the amphiphiles. It is therefore important to note that in amphiphiles, the spatial distribution—also referred to as spatial topology—of the hydrophilic and hydrophobic segments plays a critical role in determining the type of supra-structures that these can form via self-assembly [62,63]. In this context, one can differentiate between strongly amphiphilic systems, which are molecules whose hydrophobic and hydrophilic moieties or groups are strongly segregated in space, and weakly amphiphilic systems or pseudo-amphiphiles for those molecules or polymers that are mixed at the molecular level. In addition, the topology and spatial distribution of the hydrophilic and hydrophobic functional groups determine the geometry of the supra-structures obtained [64]. This is also true, as we will see next for the JNPs [65].

Another aspect is the polarity contrast between the hydrophilic (typically the polar component) and the hydrophobic (typically the nonpolar component) of the amphiphiles. This contrast is typically gauged by the so-called hydrophilic–lipophilic balance (HLB) concept. The HLB is a semi-quantitative scale developed in the 1940s by William C. Griffin at Atlas Powder Company to describe the relative affinity of a surfactant molecule for water (hydrophilic) versus oil (lipophilic) environments. This concept allows formulators to predict and tailor the emulsifying behavior of surfactants, particularly in systems involving oil-in-water (O/W) or water-in-oil (W/O) emulsions.

For non-ionic surfactants, Griffin [66,67] defined the *HLB* number on a scale of 0 to 20 using the equation:

(1) $$HLB=20\times \frac{M_{h}}{M}$$

where *M_h_* is the molecular mass of the hydrophilic (polar) portion of the molecule and *M* is the total molecular mass of the molecule, whereas the above equation can also be adapted by replacing the mass parameter with area, area of the hydrophilic or hydrophobic part, to length, etc.

Surfactants with *HLB* values below 9 are generally more lipophilic and suitable for stabilizing W/O emulsions, while those with HLB values above 11 are more hydrophilic, favoring O/W emulsions. Values between 9 and 11 are considered borderline or transitional.

Griffin’s HLB system has since become a tool in colloid and interface science, widely used in the formulation of cosmetics, pharmaceuticals, agrochemicals, and food emulsions.

## 4. The Manifestation of Amphiphilicity—Scalability Beyond the Molecular Scale

Traditionally confined to molecular surfactants and block copolymers, recent advances in nanotechnology have demonstrated that amphiphilicity can be extended beyond the molecular scale, and JNPs are among the engineered colloidal structures that prove that amphiphilicity is scalable beyond that of molecules and polymers. These particles, named after the two-faced Roman god Janus, exhibit dual-wettability, enabling them to act as surfactants at interfaces, assemble into complex architectures, and exhibit unique physicochemical behaviors distinct from their molecular counterparts.

To prove that JNPs indeed behave like amphiphiles, they must exhibit the same type of behavior as molecular surfactants, such as the following: (i) exhibit adsorption at interfaces with a corresponding reduction in interfacial tension; (ii) stabilize emulsions; (iii) stabilize foams; (iv) self-assemble into supramolecular structures like micelles, vesicles, or bilayers, and the list can be expanded.

HNPs and JNPs do exhibit spontaneous adsorption at interfaces, and this is thermodynamically driven by maximizing the entropy of the system formed between two immiscible phases. The earlier theoretical works of Binks and colleagues [7,68] demonstrate that truly amphiphilic JNPs resemble surfactants, in that they can spontaneously adsorb at liquid–liquid interfaces, e.g., oil/water or water/air, and their interfacial activity can be several times larger than that of HNPs [7], which, as already discussed above, due to their distribution of hydrophilic and hydrophobic groups, can only at best be pseudo-amphiphilic. In the case of JNPs, the rotation and desorption at the interface is blocked due to the fact that the apolar lobe is wetted by the apolar phase and the polar phase is immersed and wetted by the polar phase.

Although amphiphilicity is a fundamental theoretical concept describing the dual affinity of a molecule or particle for polar and nonpolar environments, it currently lacks a universally accepted quantitative definition, meaning a rather relative than absolute definition. In contrast, parameters such as interfacial adsorption, interfacial activity (e.g., the ability to reduce interfacial tension), and energy of attachment to interfaces are experimentally measurable quantities that reflect manifestations of amphiphilicity in real systems. These parameters are often used as indirect indicators of amphiphilic behavior. However, despite their conceptual correlation with amphiphilicity, no direct, mathematical relationship has yet been established that quantitatively links amphiphilicity as a unified descriptor to these measurable interfacial properties. This lack of formalism presents a significant gap in the theoretical framework of amphiphilic systems, particularly at the nanoscale where complex surface anisotropies come into play. Therefore, to better elucidate how amphiphilicity governs interfacial behavior, the next section will explore in detail the individual relationships between amphiphilicity and these experimentally accessible quantities.

### 4.1. Amphiphilicity vs. Interfacial Attachment/Adsorption Energy of JNPs

As previously mentioned and well-established in practice, HNPs can spontaneously adsorb at liquid interfaces, which renders them interfacially active [13,18]. In other words, when adsorbing at a water/air or oil/water interface, the particle becomes partially wetted by both immiscible phases. But how can a single particle be wetted simultaneously by two distinct phases? This behavior arises from the fact that each liquid phase exhibits a specific physicochemical interaction with the particle’s surface. If such interactions were absent, the particle would preferentially reside entirely in one of the phases rather than localize at the interface. Therefore, even HNPs exhibit a measurable degree of amphiphilicity—or more precisely, “amphipathicity”—through their simultaneous interaction with both polar and nonpolar environments. This dual affinity is typically enabled by the presence of both polar and apolar functional groups on the particle surface, albeit not spatially segregated into distinct hemispheres. For this reason, we refer to this behavior as pseudo-amphiphilicity, distinguishing it from the topographically defined amphiphilicity observed in Janus nanoparticles.

**Figure 2:**
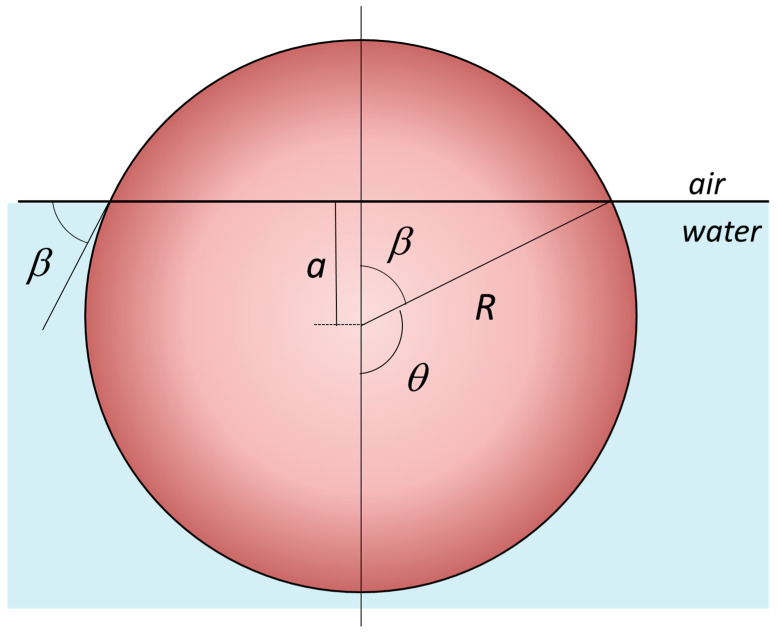
Spherical particle adsorbed at the water/air interface. The contact angle with water, *β*, and its relationship with the parameter, *a*, that describe the immersion depth of the particle in water.

The interfacial adsorption energy as an HNP function of the particle’s immersion depth “*a*” at the oil/water interface was derived by Pieranski [69], and this can be calculated from the interfacial contact angle *β*, as seen in Figure 2, where we have taken the water/air interface for simplicity [8]. Thus, the energy of attachment for a HNP, of radius *R*, at the air–water or oil–water interfaces can be calculated from the following equation [70]:

(2) $$E_{interface}=\gamma_{\left(air,water\right)}\times \pi R^{2}{\left(1\pm \cos\left(\beta \right)\right)}^{2}$$

where the expression of the interfacial immersion depth “*a*” is related to the interfacial tension via the Young–Dupré contact angle:

(3) $$\cos\beta =\frac{a}{R}=\frac{\gamma_{\left(HNP,air\right)}\pm \gamma_{\left(HNP,water\right)}}{\gamma_{\left(air,water\right)}}$$

where the $\gamma_{\left(HNP,air\right)}$, $\gamma_{\left(HNP,water\right)}$, and $\gamma_{\left(air,water\right)}\;$ are the interfacial energies of each particle interface with air and water; the sign in the parenthesis is negative for β<90° and positive for β>90°. From the above equation, the highest adsorption energy is achieved when the cosβ=0, meaning that β=90°, the HNP is immersed half-way at the interface.

**Figure 3:**
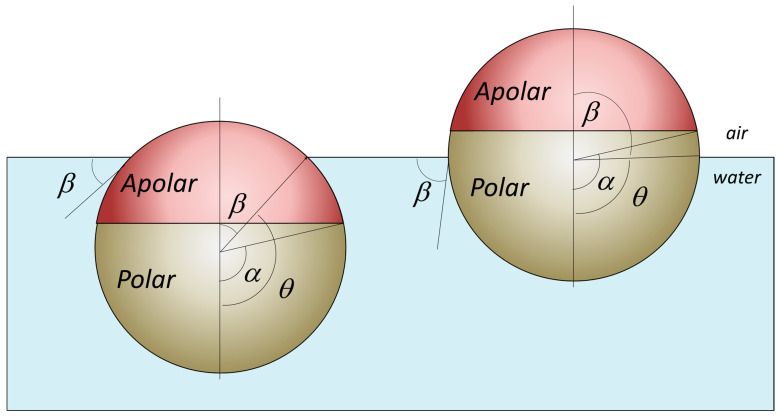
A perfectly spherical JNP adsorbed at the air/water interface. (**Left**) Case of partial de-wetting of the A-lobe, θ>α and the general case α≠π/2. (**Right**) Case of partial de-wetting of the P-lobe, θ<α and the general case α≠π/2.

Applying the same reasoning to JNPs, Binks [7,68], Ondarçuhu [71], Honciuc [8,59], and others have calculated the attachment energies for spherical JNPs, consisting of two hemispheres, one polar and one apolar, as shown in Figure 3. The expressions for the attachment energies of JNPs at interfaces are significantly more complex to model than for HNPs, and only for simple architectures and particular orientations.

For example, by taking a perfectly spherical JNP adsorbed at water/air or oil/water interfaces, consisting of two hemispheres, one polar (P) and the other apolar (A), there are two general cases, as depicted in Figure 3: (i) case of partial de-wetting of the A-lobe, *θ* > α and the general case α ≠ π⁄2; (ii) case of total de-wetting of the A-lobe and partial de-wetting of the P-lobe, *θ* < α and the general case α ≠ π⁄2. The case α ≠ π⁄2 means that the boundary between the A and P lobes is not at the interface. These expressions for detachment/adsorption free energy of the JNP depicted in Figure 3 were given by Honciuc [8]. In order to find the free energy of the particle at the interface, we must first find the expression for the interfacial energies that the particle generates in each medium. For example, for the particle in Figure 3, lobe P or lobe A are immersed in water or air; therefore, we must account the energy of each interface, namely the *E_(A,air)_*_,_ *E_(P,air)_*, *E_(A,water)_*, *E_(P,water)_*, and *E_(air,water)_* as a function of the contact angle, interfacial tension, and solvent-exposed areas of each lobe.

As an example, for case of partial de-wetting of the A-lobe, θ>α and the general case α≠π/2, as depicted in Figure 3:

(4) $$E_{\left(A,\;air\right)}=\gamma_{\left(A,\;air\right)}\times A_{\left(A,\;air\right)}=\gamma_{\left(A,\;air\right)}\times 2\pi R^{2}(1+\cos\left(\theta \right))$$

(4a) $$E_{\left(A,\;water\right)}=\gamma_{\left(A,\;water\right)}\times A_{\left(A,\;water\right)}=\gamma_{\left(A,\;water\right)}\times 2\pi R^{2}(\cos\left(\alpha \right)-\cos\left(\theta \right))$$

(4b) $$E_{\left(air,\;water\right)}=\gamma_{\left(air,\;water\right)}\times A_{\left(of\;circular\;base\;radius\;R\right)}=\gamma_{\left(air,\;water\right)}\times \pi R^{2}{sin}^{2}\left(\theta \right)$$

Thus, the total free energy of the JNP at the interface is the sum of these energies:

(5) $$E=2\pi R^{2}\times \left[\gamma_{\left(A,\;air\right)}\times \left(1+\cos\left(\theta \right)\right){+\gamma }_{\left(A,\;water\right)}\times \left(\cos\left(\alpha \right)-\;\cos\left(\theta \right)\right)+\gamma_{\left(P,\;water\right)}\times \left(1-\cos\left(\alpha \right)\right)-\gamma_{\left(air,\;water\right)}\times \frac{1}{2}{sin}^{2}(\theta )\right]$$

For the particular case of α = π⁄2 and using the relationship θ=π−β, the total free energy of the JNP at the water/air interface in terms of the contact angle *β* can be expressed as follows:

(6) $$E_{interface}=2\pi R^{2}\times \left[\gamma_{\left(A,\;air\right)}\times \left(1-\cos\left(\beta \right)\right){+\gamma }_{\left(A,\;water\right)}\times \cos\left(\beta \right)+\;\gamma_{\left(P,\;water\right)}+\gamma_{\left(air,\;water\right)}\times \frac{1}{2}{sin}^{2}(\beta )\right]$$

The expression of the free energy of a JNP at the water/air interface, in the above equation is thus significantly more complex than that of an HNP, as given in Equation (2) [72].

Binks and Fletcher [7] proposed that a particle’s interfacial activity could be assessed via its attachment/detachment energy and argue that this is a gauge of interfacial activity and therefore of the “amphiphilicity”. However, since these energies scale with *R*^2^, this approach becomes inherently size-dependent and even shape-dependent and unsuitable for comparing particles of different dimensions and morphologies. For instance, under this framework, a large object, such as a tennis ball, would appear more interfacially active than a nanoscale particle, leading perhaps to an evidently misleading conclusion. For JNPs and HNPs of the same size, the attachment energy is typically taken to be of a factor of 3 and larger for the former particles [7], but in fact, the interfacial activity of these dual particles can be significantly larger than a factor of three for other geometries, as shown in simulations by Gao et al. [49]. However, it would be more useful if amphiphilicity can be discussed quantitatively and can be measured. And, as already mentioned previously, there is currently no mathematical formalism linking the two: energy of adsorption/desorption vs. amphiphilicity, although many rely on correlative arguments [73]. We instead propose that changes in surface or interfacial tension upon particle addition serve as a more appropriate and experimentally meaningful criterion for evaluating interfacial activity. However, before proceeding to this aspect of gauging interfacial activity, another aspect must be first discussed, namely the measurement of amphiphilicity or the amphiphilic strength of a particle.

### 4.2. Amphiphilicity vs. Janus Balance and Polarity Contrast Between the Lobes

In the previous section, we have discussed the HLB concept to gauge the amphiphilicity of the surfactants. When the surfactants receive a certain value on the HLB scale, this becomes extremely important for the experimentalists, who already know which surfactant is able to stabilize oil-in-water or water-in-oil emulsions, or which are foaming agents or de-foaming agents. Therefore, a similar concept would be extremely useful for JNPs in their application and a priori evaluation of their ability to interact with the environment. For example, Lee and Yu [74] have established a connection between the JNPs hydrophilic–hydrophobic contrast and the particle’s ability to disrupt lipid membranes.

**Figure 4:**
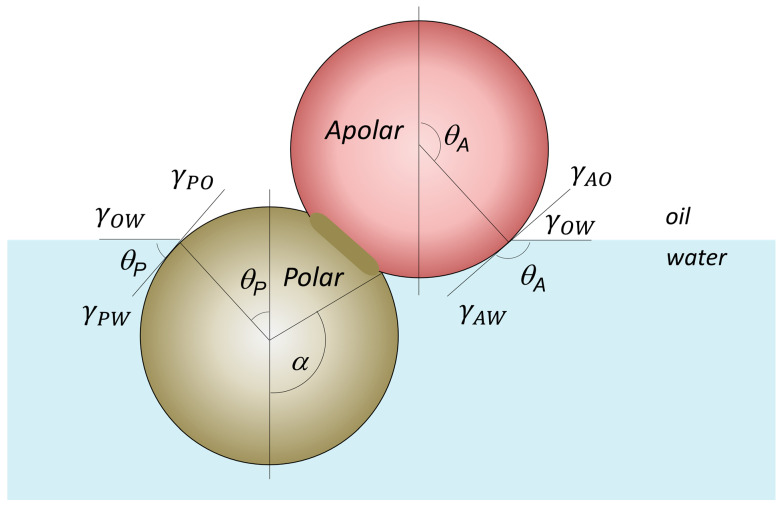
Hypothetical model describing a spherical dumbbell or snowman-type JNP, with an apolar “*A*” and a polar “*P*” lobe adsorbed at the oil–water interface. For simplicity, the two lobes have the same diameters. The *θ_A_* is the water contact angle of the apolar Janus lobe and the *θ_P_* is the water contact angle of the water to the polar Janus lobe of the JNP.

Thus, Jiang & Granick [73] have introduced the concept of Janus balance or “*J*-value” to effectively quantify the amphiphilicity as the dimensionless ratio of work to transfer of a JNP from the oil–water interface into the oil phase, normalized by the work needed to move it into the water phase:

(7) $$J=\frac{\sin^{2}\alpha +2\cos\theta_{P}(\cos\alpha -1)}{\sin^{2}\alpha +2\cos\theta_{A}(\cos\alpha +1)}$$

where the angle *α* has the same meaning as that depicted in Figure 3, which keeps track of the position of the boundary between the polar (*P*) and apolar (*A*) hemispheres of the JNPs with respect to the interface, as depicted in Figure 4. *θ_A_* and *θ_P_* are the contact angle at the interface of the apolar and polar Janus lobes as depicted in Figure 4.

Equation (7) indicates that the Janus balance *J* depends on both the relative surface areas of the hydrophilic and hydrophobic lobes *α* and on the intrinsic wettability of each lobe, described by the contact angles *θ_A_* and *θ_P_*. When *θ_A_* and *θ_P_* are held constant, *J* increases with increasing *α*, corresponding to a larger hydrophilic area (because cos *θ_P_* < 0). Conversely, if *α* is fixed, *J* increases as either *θ_A_* becomes more acute (indicating greater hydrophilicity) or *θ_P_* becomes less obtuse (indicating reduced hydrophobicity). A larger magnitude of *J* reflects a more hydrophilic character of the Janus particle, following the same qualitative trend as the HLB used to describe molecular surfactants—where a higher HLB corresponds to greater affinity for water [67]. However, this model includes two important assumptions: (i) it assumes an idealized orientation of the particle, as in Figure 3, with the Janus axis aligned perpendicular to the interface; (ii) it presumes a perfectly spherical geometry, as in Figure 3. In Figure 4, however, we have attempted to extrapolate this model to dumbbell or snowman JNPs. Therefore, while informative, the current model is not generally applicable across all Janus particle architectures.

**Figure 5:**
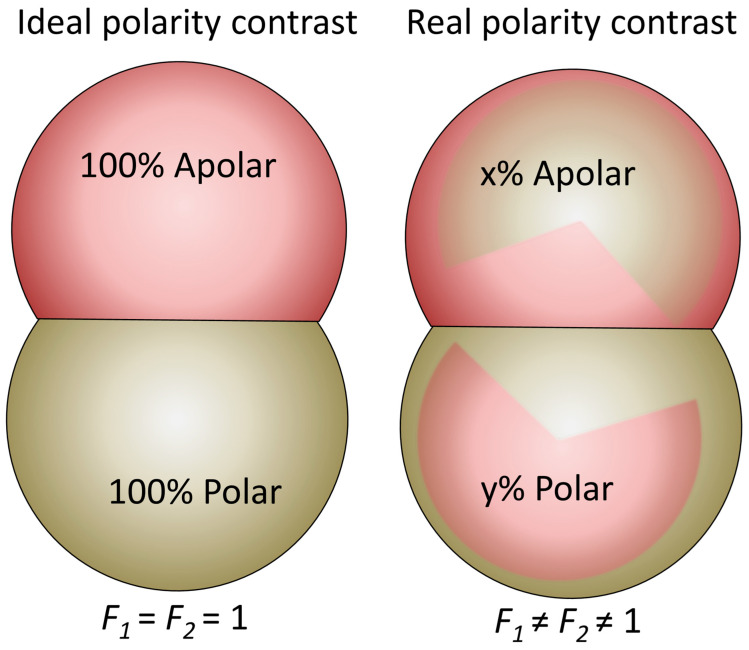
Hypothetical amphiphilic snowman-type JNP displaying an ideal polarity contrast *F*_1_ = *F*_2_ = 1 vs. a more realistic representation of a snowman Janus particle with the surface polarity of the lobes departing from ideality *F*_1_ ≠ *F*_2_ ≠ 1.

On the other hand, Honciuc et al. [1,3,8,75] have proposed the direct calculation of the HLB of a JNP using a modified Griffin’s approach [66] for JNPs:

(8) $$HLB=20\times \frac{A_{Polar}\times F_{1}}{A_{Polar}\times F_{1}+A_{Apolar}\times F_{2}}$$

where the $A_{Polar}$ is the area of the polar lobe, $A_{Apolar}$ the area of the nonpolar lobe, and, in addition, we have introduced *F_i_*, _(*i* = 1,2)_—weighing factors accounting for the “degree” of polarity of the lobes. The above equation takes the value of 20 for *F*_2_ = 0 and 0 for *F*_1_ = 0, which are two limiting situations: strongly polar and apolar particles, with no amphiphilicity. On the other hand, a value of *F*_1_ = 1 and *F*_2_ = 1 assumes an “ideal” polarity contrast between the two surface regions, as seen in Figure 5, and thus the HLB are determined only by the geometry of the lobes, i.e., their aspect ratio [8]. Honciuc [8] proposed that the polarity weighting factors *F* can be calculated from the ratio between the polar and apolar or dispersive surface energy components for each of the Janus lobes, as seen in Figure 5:

(9) $$F_{1}=\frac{\gamma_{1}^{p}}{\gamma_{1}^{p}+\gamma_{1}^{d}},\;\mathrm{and}\;F_{2}=\frac{\gamma_{2}^{d}}{\gamma_{2}^{p}+\gamma_{2}^{d}}$$

where the small Greek gammas are the surface energies, and the superscripts “*p*” and “*d*” indicate the polar and dispersive or apolar surface energy components of the corresponding Janus lobes, 1—polar lobe and 2—apolar lobe. In practice, to determine *F*, one must know the surface energy and its polar and dispersive components, which is not trivial.

This has turned out to be a more useful approach for experimental evaluation of the Janus balance than the one mentioned before. For example, Mihali & Honciuc [1] have measured the surface energy and the polar/disperse components of each Janus lobes in a homologous series of semiconducting polypyrrole/poly(3-(trimethoxysilyl)propyl methacrylate) PPy/P(3-TSPM) JNPs with increasing size of the polar lobe, and with these values, they have calculated the corresponding weighting factors *F* and, subsequently, the HLB values, which are given in Table 1. Interestingly, the HLB values calculated for the JNPs without applying weighting factors (Table 1, column 5) are similar to those obtained when accounting for the polarity of the lobes (Table 1, column 8). This consistency arises because the measured *F*-values are close to unity, indicating that the JNPs exhibit an almost-ideal polarity contrast. The weighting factors used in these calculations were derived from the polar and dispersive components of the surface energy, determined using the Owens–Wendt–Rabel–Kaelble (OWRK) method [59]. This method requires contact angle measurements with at least two or three test liquids of known surface tension, each having distinct polar and dispersive components. However, Shuting Xie et al. [76] have used the same equation as above to calculated the Janus balance for homologous series of snowman-type poly(styrene-co-acrylic acid)/polystyrene (CPSAA/PS) JNPs with varying lobe size ratio, considering the values of *F*_1_ = 1 and *F*_2_ = 1 (with an “ideal” polarity contrast between the two lobes) and obtained the values in Table 1.

Quantifying the HLB of JNPs using a surface polarity contrast between the lobes by a weighted surface energy approach provides a useful predictive tool for evaluating their emulsification behavior. When applied across a homologous series of JNPs, this method allows for systematic insights into their role as emulsifiers of O/W or W/O systems. Expressing amphiphilicity on the established Griffin HLB scale (ranging from 1 to 20) makes the results easily interpretable, especially for researchers familiar with conventional surfactants. On this scale, JNPs with HLB values below 10 tend to stabilize W/O emulsions due to stronger affinity for the oil phase, whereas those with values above 10 are more effective as O/W emulsifiers—preferences that were clearly confirmed by our emulsification experiments [3,41]. This approach offers a significant advantage over geometric or orientation-based models of Janus balance, as it does not rely on assumptions about particle shape, interfacial orientation, or positioning. It is applicable regardless of whether the particles are spherical, anisotropic, or irregularly shaped, making it a universally adaptable method for quantifying amphiphilicity in colloidal systems.

To conclude, the ability to express JNP amphiphilicity on a conventional HLB scale bridges molecular and nanoscale amphiphiles within a unified framework, enabling broader comparison and even functional prediction.

### 4.3. Amphiphilicity vs. Interfacial Tension Reduction

As already stated, the defining feature of surfactants and amphiphilic molecules is their ability to adsorb at liquid–liquid or liquid–air interfaces and, upon adsorption, to reduce the interfacial or surface tension. Mechanistically, this process involves the hydrophobic moiety of the amphiphile—initially solvated in water—undergoing partial or complete de-wetting from the aqueous phase, followed by rewetting by the nonpolar phase (such as oil) or exposure to air. Numerous studies have demonstrated that amphiphiles with hydrophobic alkyl chains, such as fatty acids, spontaneously orient at the water surface, forming ordered monolayers in which the hydrophobic tails protrude from the water due to de-wetting effects.

Despite extensive experimental evidence, no rigorous theoretical framework currently exists that quantitatively links a specific molecular parameter of a surfactant to the extent of surface tension reduction it induces [8,59]. Nonetheless, the general mechanism can be rationalized qualitatively. When surfactants accumulate at the interface, they disrupt the strong cohesive “lateral” interactions—primarily hydrogen bonding and van der Waals forces—between water molecules in the interfacial layer. These are replaced by weaker interactions among the hydrophobic tails (primarily van der Waals) and, in the case of ionic or polar headgroups, by additional electrostatic repulsions between neighboring molecules. This replacement of cohesive water–water interactions with less cohesive surfactant–surfactant interactions leads to a net decrease in surface or interfacial tension.

In fact, for surfactants bearing long, saturated alkyl chains, the reduction in the surface tension of water tends asymptotically toward the surface tension of liquid alkanes with chain lengths matching those of the surfactant tails. This observation underscores the dominant role of the alkyl chain–air or alkyl chain–oil interface in governing the final surface energy once the monolayer is fully established [77,78,79].

A similar mechanism operates in the case of JNPs. Upon interfacial adsorption, the amphiphilic nature of the JNPs leads to anisotropic wetting: the hydrophilic lobe remains immersed in water, while the hydrophobic lobe undergoes de-wetting from the aqueous phase and is re-wetted by the nonpolar phase (e.g., oil or air). This asymmetric orientation at the interface is energetically favorable and reduces the system’s Gibbs free energy. One key contributing factor is the release of structured water molecules—often forming clathrate-like hydration shells—surrounding the hydrophobic surface. When the hydrophobic lobe exits the aqueous phase, these solvent molecules are liberated, leading to an increase in the entropy of the system, which in turn lowers the overall free energy [59].

Upon adsorption, JNPs also lose rotational freedom as they acquire a preferred orientation at the interface, further contributing to the stabilization of the interfacial structure. Numerous experimental studies have reported a measurable reduction in interfacial tension upon adsorption of amphiphilic JNPs, affirming their role as effective interfacial stabilizers [1,2,3,4].

For soft JNPs, or those composed of polymeric coronas or polymer brushes grafted onto solid particles, the mechanism of interfacial adsorption and reduction in surface tension closely parallels that of conventional molecular surfactants: polymer chains extend into both phases, disrupting cohesive forces at the interface and thereby lowering interfacial tension [80]. In contrast, for rigid, solid-state JNPs that adsorb as discrete colloids and form dense interfacial monolayers, the mechanism is more complex. At a certain size and morphology, these particles could also generate interfacial menisci, and the curvature of these menisci—along with capillary interactions—plays a critical role in determining the net reduction in interfacial tension [81,82,83]. As particle size increases, the formation of interfacial menisci becomes more prominent in governing lateral interactions, unlike molecular amphiphiles, which do not induce such effects. This phenomenon highlights the scalability of amphiphilicity, manifesting at larger scales where it begins to compete with macroscopic forces, such as gravitational, drag, capillarity, buoyancy, etc. Additionally, the physical presence of JNPs at the interface displaces water molecules, thereby replacing strong water–water-cohesive interactions with weaker water–particle-adhesive interactions.

Despite these qualitative insights, a comprehensive theoretical framework is still lacking. A mathematical formalism that quantitatively relates the structural, chemical, and geometrical features of JNPs to their ability to reduce interfacial tension is needed. Such a model would significantly advance our understanding of colloidal amphiphiles and enable predictive design of interfacial materials.

**Figure 6:**
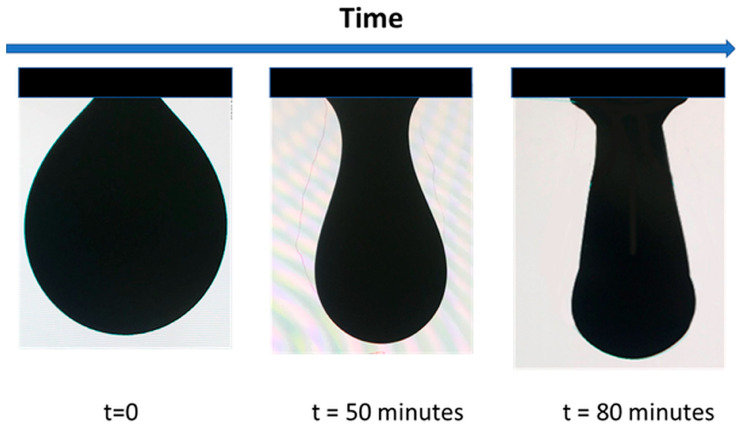
Pendant drop tensiometry: images of an elongated pendant drop showing visually the reduction in surface tension by a larger deformation of the droplet from that of pure water at t = 0. Reproduced with permission from [16], Copyright © 2021 Wiley-VCH GmbH.

The reduction in interfacial tension is a measurable effect using standard surface tension methods, most typically by pendant drop tensiometry, as depicted in Figure 6, but the classical Wilhelmy plate methods are also applicable. Figure 6 depicts the elongation of a water droplet containing JNPs over time, indicating the reduction in the surface tension.

For example, solid-state amphiphilic Janus silica nanoparticles reduced oil–water interfacial tension to as low as 2.28 mN/m at 0.05 wt% concentration, while also altering rock wettability and forming stable interfacial films beneficial for EOR applications [84]. Meanwhile, Giraldo et al. [85] have demonstrated a maximum decrease in the interfacial tension between water and crude oil by 34% in the presence of NiO/SiO_2_ JNPs and only a 21% for SiO_2_ HNPs at a concentration of 100 mg/L, leading to a 50% enhancement for the JNPs and no enhancement in EOR for HNPs at the same concentration. These latter studies demonstrate the difference in interfacial activity for JNPs vs. HNPs. Similar recent studies have been performed by Cao et al. [86] and Liu et al. [87].

Several literature results are compiled in Table 2 and the reported values highlight the extent of tension reduction as a function of JNP concentration and composition, demonstrating their effective interfacial activity compared to homogeneous or pseudo-amphiphilic particles.

The data in Table 2 presents compelling evidence that solid-state amphiphilic JNPs are capable of significantly reducing surface and interfacial tensions at liquid–liquid and liquid–air interfaces. Three main classes of JNPs are represented as follows: (i) silica-based Janus particles with asymmetric surface functionalization, (ii) polymeric Janus nanoparticles synthesized via seeded emulsion polymerization, and (iii) metallic or inorganic JNPs functionalized with polar and apolar ligands. Silica JNPs obtained by functionalizing each hemisphere with distinct chemical groups (e.g., oleic acid, HMDS/APTS, octyl/amino groups) consistently showed marked interfacial activity. For instance, Saeedi Dehaghani et al. [88,89] demonstrated that CH_4_ foam stabilized with such particles resulted in up to 25 mN/m greater reduction than homogeneous SiO_2_. Similarly, Tang et al. [90] reported a striking decrease in surface tension from 69.8 to 36.4 mN/m for water–air systems using 0.05 wt% JNPs, and even more impressively, a drop to 0.067 mN/m in oil–water systems—indicating near-complete elimination of cohesive forces at the interface. Polymeric JNPs (e.g., PS–PDIPAEMA/P(3-TSPM)) also demonstrated superior performance compared to their homogeneous counterparts, reducing interfacial tension in toluene–water and heptane–water systems by over 10 mN/m, as shown by Wu and Honciuc [92]. Interestingly, surface tension reduction varied depending on the oil phase, indicating sensitivity to the chemical environment and possible tuning of amphiphilicity for targeted applications.

The results show that true amphiphilicity, where the two hemispheres are chemically distinct and spatially segregated, leads to more efficient interface stabilization compared to pseudo-amphiphilic or homogeneous particles. This is evident not only from numerical values but also from mechanistic insights: Janus particles reduce interfacial tension by orienting at the interface, replacing strong cohesive water–water interactions with weaker particle–liquid adhesion forces, and generating favorable entropy changes due to release of structured water [8,59].

However, despite the demonstrated ability of amphiphilic Janus nanoparticles to reduce surface and interfacial tension, this continues to remain a topic of interest for researchers. This is primarily because the interfacial behavior of JNPs—measurable by interfacial tension reduction—is strongly influenced by the particle’s morphology, surface chemistry, material hardness, contrasting wettability, scale-dependent interaction forces, and other structural design parameters, which are not encountered at the molecular scale of surfactants. Linking all these variables into a single formalism is an almost impossible task. Yet, elucidating case-by-case the underlying mechanisms responsible for surface tension reduction by JNPs of various designs may uncover new scientific insights and nanomaterials with engineered interfacial properties, from which future practical applications may greatly benefit.

In conclusion, the systematic reduction in surface and interfacial tension observed for solid-state amphiphilic JNPs across multiple systems and chemistries demonstrates their robust interfacial activity. These results validate their application as next-generation colloidal surfactants and justify further theoretical efforts to quantitatively model their behavior at complex interfaces

### 4.4. Amphiphilicity vs. Self-Assembly

Amphiphilicity not only governs the interfacial activity of JNPs but also drives their ability to self-assemble into supra-structures. Analogous to molecular amphiphiles, amphiphilic JNPs exploit their dual affinity to spontaneously organize into a variety of configurations depending on the surrounding environment, interfacial geometry, and particle–particle interactions. This section explores how the amphiphilic character of JNPs underlies their ability to form micelles, capsules, vesicles, foams, emulsions, and interfacial monolayers. Such assemblies are critical for advanced material design, encapsulation, and interfacial engineering.

#### 4.4.1. Formation of Supra-Structures in Homogeneous Media: From Micelles to Capsules and Vesicles

In homogeneous media like water, individually dispersed amphiphilic JNPs—similar to surfactants—can self-organize into micellar, vesicular, or capsule-like architectures, either in bulk phases or at interfaces. These structures arise from directional interactions to minimize interfacial energy, where JNPs orient such that their hydrophilic and hydrophobic domains interact favorably with other particles. Unlike molecular surfactants that produce soft self-assembled structures, however, the rigid geometry and anisotropic surface chemistry of JNPs can lead to discrete and structurally resistant and persistent aggregates with programmable sizes and morphologies. Anisotropic architectures in colloidal systems have been extensively reviewed in recent works [101].

**Figure 7:**
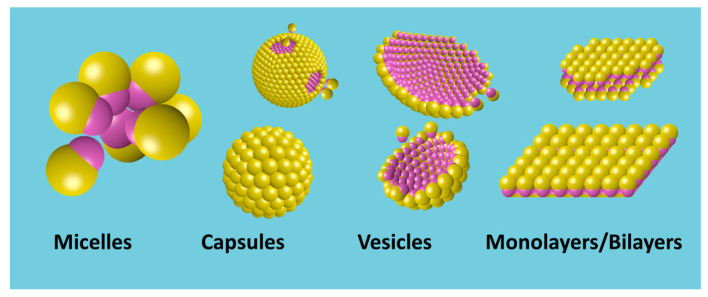
Schematic representation of the self-assembly JNPs into structures with decreasing curvatures, from micelles into capsules, vesicles, and monolayers.

This self-assembly behavior is driven by the anisotropic surface properties of JNPs, leading to the formation of structures such as micelles, vesicles, and capsules, as depicted in Figure 7, which result from directional Janus lobe—Janus lobe interactions, which can be attractive, as well as hydrophobic, van der Waals, and repulsive double-layer interactions. These self-assembled architectures are of interest for applications in drug delivery, sensing, and nanoreactor design [11,102,103,104].

*Self-Assembly Mechanisms and Structural Diversity:* The self-assembly of JNPs is influenced by several factors, including particle geometry, surface chemistry, and environmental conditions. For instance, Kang and Honciuc [17] have convincingly demonstrated that polymeric, snowman-shaped amphiphilic PtBA/P(3-TSPM) JNPs (~200 nm) could self-assemble into various structures, including micelles, worm-like chains, mini-capsules, and vesicles, by manipulating kinetic parameters during assembly.

**Figure 8:**
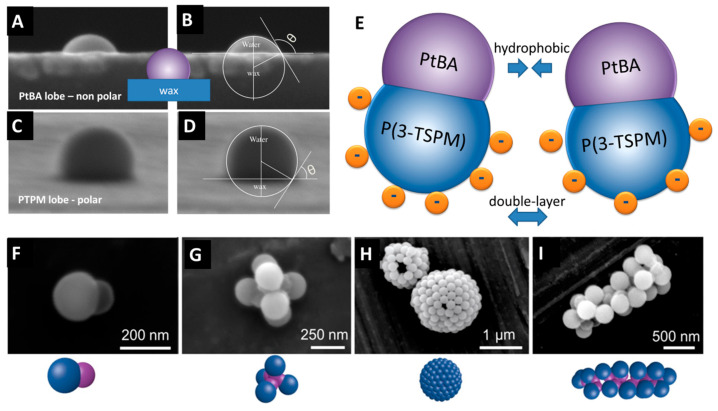
(**A**–**D**) SEM images showing the contact angle of the PtBA and P(3-TSPM) HNPs at the paraffin/wax interface. (**E**) Hypothesis of unidirectional interaction between the PtBA and P(3-TSPM) JNP lobes—attractive between the PtBA and repulsive between the P(3-TSPM) lobes—leading to self-assembly in supra-structures. (**F**,**G**) The types of self-assemblies formed, consisting of (**F**) a single JNP; (**G**) micelles with oriented JNPs, such that the PtBA hydrophobic lobe is oriented towards the inside of the structure. (**H**) Formation of mono-walled spherical capsules, and (**I**) formation of worm-like micelles or rods. (**A**–**D**,**F**,**G**) reproduced with permission from [17], Copyright© 2018 American Chemical Society.

Figure 8 captures the underlying directional interactions between amphiphilic JNPs, ca. 200 nm in diameter, synthesized from poly(tert-butyl acrylate) (PtBA) nanoparticle seeds and seeded emulsion polymerization and phase separation of a second lobe poly(3(triethoxysilyl)propyl-methacrylate (P(3-TSPM)) to generate PtBA/P(3-TSPM) JNPs. Figure 8A–D highlights the difference in wettability between the two lobes of the JNPs, evidenced by contact angle measurements at the water/paraffin wax interface. By adsorbing PtBA or P(3-TSPM) HNPs at the molten wax/water interface, then solidifying the wax and imaging by SEM, the contact angles were precisely determined. The PtBA lobe exhibits a high contact angle (Figure 8B), confirming its hydrophobic character, while the P(3-TSPM) lobe shows a lower contact angle (Figure 8D), indicating that it is the hydrophilic lobe. This asymmetric wettability is the physical foundation of the amphiphilicity in these solid-state JNPs. Figure 8E schematically depicts the inter-particle interactions forces responsible for JNP self-assembly in aqueous environments. The key attractive interactions arise between the hydrophobic PtBA lobes via van der Waals and hydrophobic attraction forces. In contrast, the hydrophilic P(3-TSPM) lobes, bearing hydrophilic and charged ions functional groups, exhibit repulsive electrostatic interactions due to the overlap of electrical double layers. This balance between directional attraction and repulsion leads to controlled, anisotropic assembly pathways.

However, while self-assembly was initially described in the chosen example in terms of anisotropic wetting and amphiphilic balance, it is important to recognize that these phenomena arise from the interplay of multiple interparticle forces. These include long-range, pairwise interactions—electrostatic repulsion via the electric double layer and van der Waals attraction—as captured in the classical Derjaguin–Landau–Verwey–Overbeek (DLVO) theory, as well as short-range non-DLVO forces. Among the latter, hydrophobic interaction is especially significant, acting over distances <2 nm and driving the close-range association of PtBA lobes. Steric repulsion, relevant in systems with grafted polymers or densely packed ligands, introduces contact-dependent repulsion that influences particle spacing and structural geometry in dense phases. The observed vesicle or capsule stability is therefore the result of a complex balance between hydrophobic attraction, van der Waals forces, electrostatic repulsion, and steric effects. Distinctively, in JNPs, these pairwise forces are spatially localized and chemically distinct on each hemisphere, unlike in HNPs. This asymmetry imposes directional constraints and entropic penalties, particularly in terms of rotational freedom, which in turn plays a crucial role in defining the geometry and architecture of the resulting supra-structures.

Figure 8F–I depicts a variety of JNP supra-structures driven by this amphiphilic architecture. Figure 8G shows a micelle-like structure where JNPs are oriented such that the hydrophobic PtBA lobes are shielded from the aqueous phase, being buried in the interior of the aggregate. This assembly minimizes the interfacial free energy, closely mimicking molecular surfactants that aggregate to hide their alkyl chains from water. Analogous structures are well documented in surfactant systems, where spherical micelles form above the critical micelle concentration (CMC) due to the hydrophobic effect [59].

Figure 8H displays single-walled capsules comprising JNPs arranged with high curvature. The formation of such monolayer capsules is particularly noteworthy and reminiscent of unilamellar vesicles formed by phospholipid amphiphiles. While double-walled (multilamellar) vesicles are common in surfactant systems, there are examples of unilamellar capsule formation using block copolymers and surfactants, especially under dilute or charge-stabilized conditions [105,106]. However, the rigidity of solid JNPs gives rise to well-defined and stable monolayer capsules, distinguishing them from soft amphiphilic systems. Finally, Figure 8I illustrates worm-like aggregates, wherein the JNPs align linearly with hydrophobic PtBA lobes forming the core and the hydrophilic P(3-TSPM) lobes exposed to the solvent. These anisotropic assemblies are analogous to worm-like micelles or cylindrical micelles formed by surfactants and amphiphilic block copolymers in water, where curvature restrictions and packing parameters favor elongated structures over spherical ones [107,108].

The critical interplay and the necessity of the equilibrium between repulsive and attractive forces in the self-assembly of the JNPs was also emphasized by the computational studies of Granick et al. [109], by Borówko and colleagues [110,111,112], and others.

Similarly, Liu et al. synthesized Janus dumbbell nanocrystals composed of gold and iron oxide Au−Fe_3_O_4_ JNPs (<20 nm), which self-assembled into clusters, chains, vesicles, and capsules by tuning the Janus balance and assembly conditions [113]. The Fe_3_O_4_ lobe was rendered hydrophilic through ligand exchange with 3,4-dihydroxybenzoic acid, forming stable five-membered chelate rings that introduced carboxyl surface groups, thus imparting negative surface charge and colloidal stability in aqueous media. Conversely, the Au lobe remained hydrophobic due to functionalization with oleylamine or alkanethiols via Au–S bonding. This anisotropic functionalization induced a directional interplay of attractive van der Waals and hydrophobic interactions between the Au lobes and repulsive electrostatic forces between Fe_3_O_4_ hemispheres.

Crucially, Liu et al. [113] demonstrated that the degree and geometry of self-assembly into worm-like or vesicular aggregates could be modulated by tuning the so-called Janus balance. This was achieved via (i) variation of the Fe_3_O_4_/Au size ratio, (ii) selection of hydrophobic ligands of different lengths for the Au domain, (iii) pH-mediated tuning of surface charge density on Fe_3_O_4_, etc. These strategies enabled the control of amphiphilic contrast and interparticle interactions, ultimately steering the formation of one-dimensional chains, rings, or two-dimensional membranes. Their findings highlight the importance of size asymmetry and interfacial ligand chemistry in directing supramolecular organization of inorganic JNPs.

Hu et al. [114] reported synthesis self-assembly of Au-organosilica JNPs (~160 nm) by manipulating the interparticle forces, e.g., van der Waals force and electrostatic force, leading to dimers and trimers.

Kirillova et al. [115] have investigated ability of solid-state JNPs obtained by asymmetric functionalization on each hemispheres with a corona of hairy hydrophilic/hydrophobic polymers, which can be positively or negatively charged, to form supra-structures via self-assembly by tuning the same type of forces, attractive van der Waals, and electrostatic repulsion.

Gao et al. [116] demonstrated that Janus micromotors functionalized with hydrophobic octadecyltrichlorosilane (OTS) coatings on one hemisphere and a catalytic Pt layer on the other, self-assembling into dynamic supra-structures via hydrophobic interactions. These assemblies display directional motility driven by hydrogen peroxide decomposition. Importantly, the motors can incorporate non-catalytic, hydrophobic particles into their assemblies, forming motor–cargo complexes capable of oriented directional motion. The structure and dynamics of the assemblies are governed by the spatial distribution of the hydrophobic and catalytic surfaces, enabling real-time structural adaptation and cargo loading. This approach offers a controlled platform for active transport and delivery of particulate cargo, with direct implications for microscale delivery systems, drug transport, and even nanorobotics.

Much larger JNPs (40 to 100 μm) prepared by microfluidics methods also appear to manifest a tendency for self-assembly, due to directed interaction. The JNPs obtained by Nie et al. [117] were amphiphilic: the contact angles of water on polymer films constituting each Janus lobe were 116.5° and 57.1°, respectively. These JNPs formed clusters with an aggregation number determined by the ratio of volume fractions of the hydrophilic and hydrophobic parts in the microbeads.

*Influence of Particle Geometry and Surface Chemistry:* The geometry of JNPs significantly affects their self-assembly behavior. Numerous computer studies anticipated the experimental ones showing the possibility of self-assembly and the variety of geometries that can be obtained via function of the particle shape [118,119,120,121]. Whitelam and Bon used computer simulations to study amphiphilic, peanut-shaped nanoparticles, revealing that these particles could form clusters, bilayers, and micelles, depending on the packing arrangements [122]. The study emphasized that particle shape and the distribution of amphiphilic regions are critical determinants of the resulting supra-structure.

Experimental results confirmed the influence of JNPs’ geometries on the type of self-assembly structures [65,113] and that the type of structures does follow the geometrical principles outlined for amphiphilic systems [108,123]. Kraft et al. [124] have combined the experimental and the theoretical findings, confirming similarities with the molecular amphiphiles.

**Figure 9:**
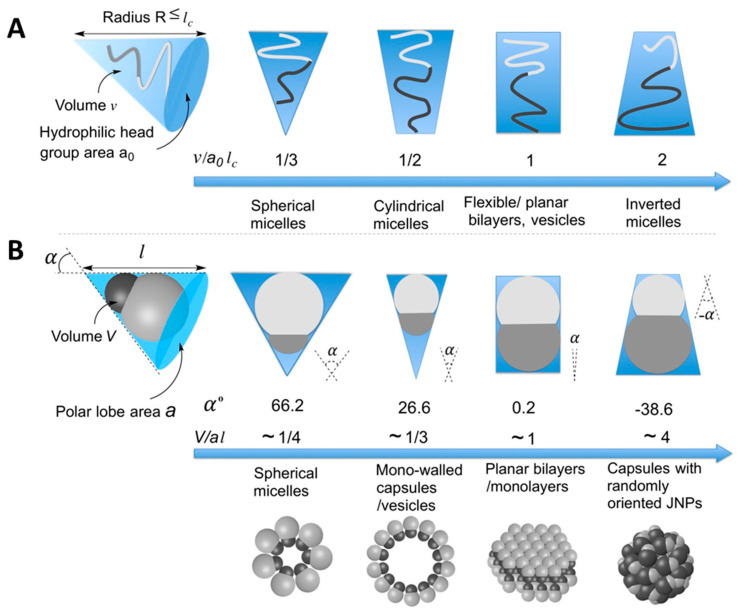
Comparison between surfactants and JNPs: (**A**) The critical packing parameter - *C_pp_ = v/a_0_l_c_* with the idealized surfactant shapes for different self-assembled supra-structures. (**B**) Representation of the Janus critical packing parameters *JC_pp_ = V/al*. The dashed lines in the figure, describe the angle *α* that is measured from the actual dimensions of the particles. Note that *l* is the height of the circumscribing structure. The double-pointed arrows indicate the dimensions of the structure. Reproduced with permission from [65], Copyright© 2018 American Chemical Society.

Kang and Honciuc [65] systematically investigated how the geometry and size ratio of snowman-type PtBA/P(3-TSPM) JNPs affect their self-assembly behavior in aqueous media. By synthesizing JNPs with varying relative lobe size ratios and controlling the surface functionalities asymmetrically, Kang and Honciuc emphasized the critical role of geometric parameters—specifically directionality (*α*) and aspect ratio (*γ*)—in dictating the self-assembly behavior of snowman-shaped JNPs, as shown in Figure 9. The driving force for supra-structure formation was identified as the hydrophobic interaction between the PtBA lobes, with self-assembly being further modulated by steric constraints and inter-particle orientation. When the non-attractive, repulsive, P(3-TSPM) lobe was larger than the PtBA lobe, the resulting high *α* value limited rotational freedom and defined bonding vectors, promoting the formation of highly oriented, anisotropic assemblies. As *α* decreased, JNPs formed low-curvature single- and double-layered sheets with diminished orientational constraints, as seen in Figure 9. Below a critical threshold (*α* < 0), particles still formed supra-structures such as capsules but increased rotational degrees of freedom introduced disorder. Furthermore, the study demonstrated that the aspect ratio *γ*, governed by the extent of phase separation during synthesis, significantly impacts self-assembly propensity. JNPs with lower *γ* values—i.e., more compact geometries—exhibited enhanced ability to form well-organized supra-structures. These findings underscore that beyond chemical anisotropy, geometric asymmetry critically modulates both interaction anisotropy and the packing geometry of JNPs in solution, enabling tunable design of colloidal amphiphiles.

**Figure 10:**
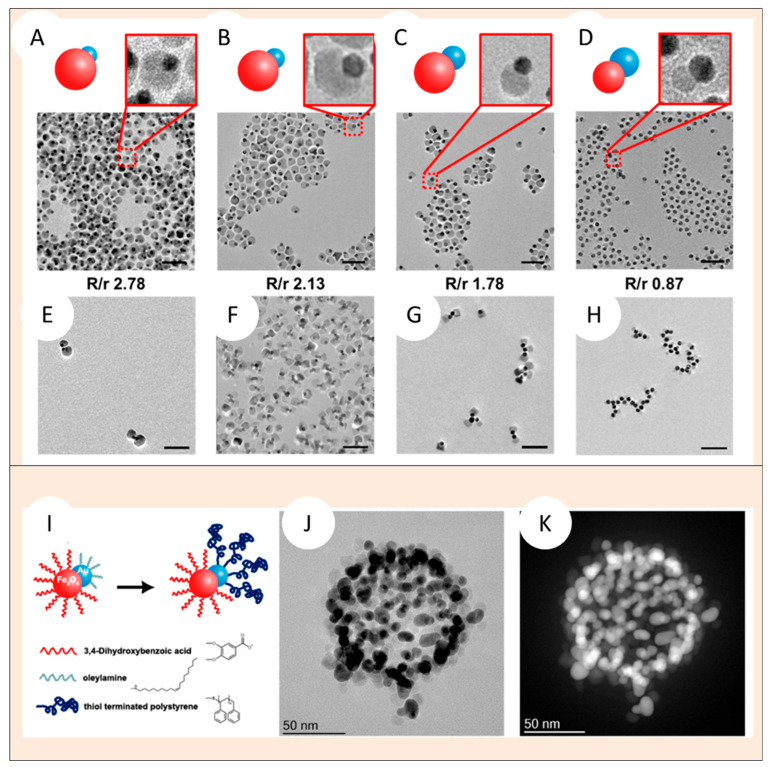
Effect of geometry and surface chemistry on the self-assembly behavior of Au–Fe_3_O_4_ Janus dumbbell nanocrystals. TEM images of Au–Fe_3_O_4_ nanoparticles with varying Fe_3_O_4_/Au lobe size ratios (R/r = 2.78 to 0.87) before (**A**–**D**) and after (**E**–**H**) surface ligand exchange. Before ligand exchange (**A**–**D**) the JNPs show random orientation and lack of directional assembly, regardless of size ratio. After ligand exchange (**E**–**H**), directional assembly emerges, driven by hydrophobic and van der Waals interactions between the Au Janus lobes. A progressive decrease in R/r enhances Au–Au contact, resulting in the formation of dimers, trimers, tetramers, and eventually linear chains as the hydrophobic Au domain increases. (**I**) Schematic of ligand exchange strategy: 3,4-dihydroxybenzoic acid on Fe_3_O_4_ and thiol-terminated polystyrene on Au, replacing oleylamine. (**J**) TEM and (**K**) HAADF-STEM images of vesicle-like aggregates formed by polystyrene-coated dumbbell nanocrystals (Mn ≈ 10,000 g/mol), in which Au lobes cluster internally and Fe_3_O_4_ lobes are exposed externally. These results highlight how both geometric lobe ratio and surface functionalization govern Janus balance and self-assembly outcomes. Reproduced with permission from [113], Copyright© 2019 American Chemical Society.

Liu et al. [113], using Au–Fe_3_O_4_ JNPs, also demonstrated that the geometry of Janus dumbbell nanocrystals exerts a decisive influence on their self-assembly behavior. By systematically tuning the Fe_3_O_4_/Au lobe size ratio from 2.78 to 0.87, they revealed that larger hydrophobic Au lobes significantly enhance inter-particle attraction and promote formation of larger clusters and one-dimensional chains. At high ratios (2.78), monomers and occasional dimers dominate, whereas moderate ratios (~2.13) yield increased dimers, trimers, and tetramers, as seen in Figure 10A–H. Further reducing the ratio to 1.16 and 0.87 leads to pentamers and even long chains, suggesting that hydrophobic core size drives assembly magnitude by providing greater van der Waals surface area. Furthermore, to make the Au Janus lobe even larger—i.e.—decrease the Fe_3_O_4_/Au lobe size ratio—and increase the hydrophobic interaction strengths, the authors have grafted thiol-terminated PS brushes of increasing molecular weight. Thus, at higher molecular weights (~10 kDa), vesicle-like assemblies emerged, with Au oriented towards the inside of the structure and Fe_3_O_4_ lobes oriented towards the outside of the structure, as shown in Figure 10I–K. These structures resemble the vesicle like the self-assembled structures formed by surfactants.

Fujii et al. [15] also have shown that polystyrene-grafted Au-SiO_2_ JNPs (PS-g-Au-SiO_2_) (~5.8 nm) were able to self-assemble into micelle-like structures at JNP concentrations of 1 and 2 wt% in water driven by the hydrophobic interaction between the PS-Au lobes. However, the aggregation number of the micelles was not well-defined, in contrast to that of molecular surfactants.

Triblock JNPs also exhibit significant potential towards self-assembly, generating specific and characteristic superstructures. For example, Chen et al. [125,126] demonstrated that triblock Janus colloids—spheres with hydrophobic poles and a charged equator—can self-assemble into 2D kagome lattices. In aqueous solution, the addition of NaCl screened electrostatic repulsion, allowing short-range hydrophobic attraction between poles. Assembly followed a two-step pathway: initial formation of short strings and triangular clusters, followed by rearrangement into crystalline kagome domains. The final structure was a polycrystalline monolayer stabilized by directional, anisotropic interactions. This work shows that patch geometry and surface functionalization alone can direct the formation of ordered supra-structures without the need for lock-and-key interactions.

Kang and Honciuc [127] have also demonstrated the ability of triblock PAA−PTPM−PTMPT JNPs, obtained via seeded emulsion polymerization, into a variety of superstructures, such as spherical or wormlike micelles.

The anisotropic shape and surface properties of the Janus spheroids facilitated the formation of encapsulating structures, offering insights into designing JNPs for targeted delivery applications.

#### 4.4.2. Templated Self-Assembly at Liquid/Air Interfaces: Foam Lamellae

Amphiphilicity drives the interfacial adsorption; therefore, the amphiphilic JNPs adsorb at interfaces such as liquid/gas or liquid/air, until the surface is completely saturated, similar to the behavior of molecular amphiphiles. Upon gas sparging or agitation of a colloidal dispersion of amphiphilic JNPs, these can stabilize the gas bubbles transiting the liquid leading to the formation and stabilization of foam. In particular, foam lamellae represent a dynamic yet structured environment where amphiphilic JNPs can undergo guided interfacial assembly. In contrast to molecular surfactants, which adsorb and lower surface tension rapidly, due to their small size and fast diffusion, JNPs can form highly ordered, mechanically robust structures at the liquid/air interface, which persist even after water drainage. This could have practical implications, especially in designing porous membranes for air filtration applications.

**Figure 11:**
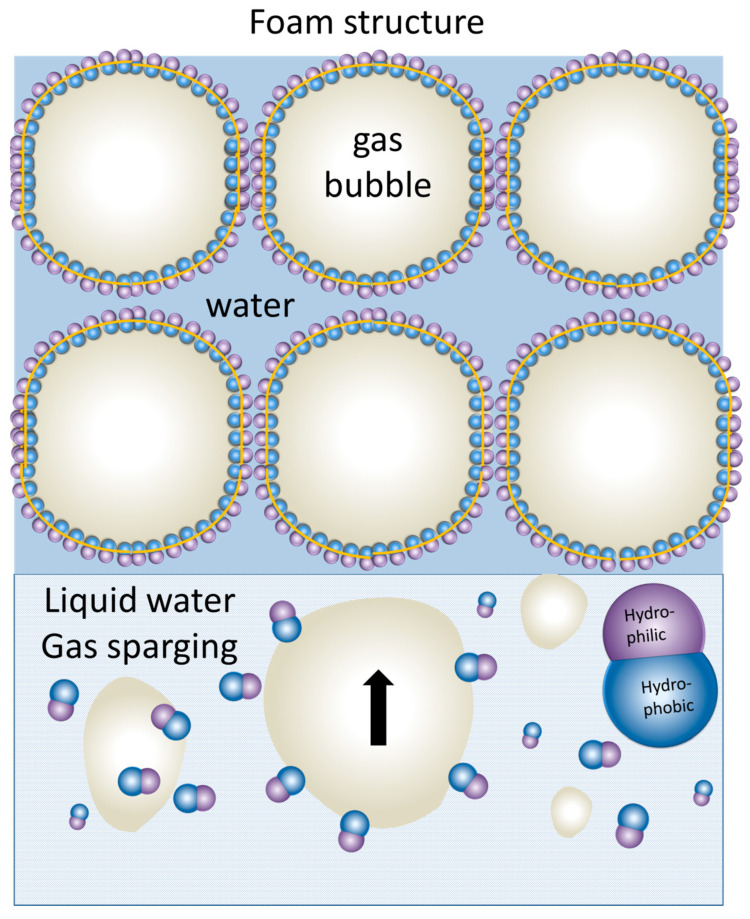
Cartoon representing the structure of the foam lamella stabilized by highly oriented, self-assembled amphiphilic JNPs. The arrow indicates the upward motion of the gas bubble, which pulls the JNPs adsorbed at the interface, to the surface of the liquid, as foam.

Structurally, the foam lamellae formed by molecular surfactants should consist of a double layer of molecules oriented such that the hydrophobic tail is oriented towards the gas and the polar head towards the water. The head-to-head arrangement maintains the water in the foam lamella, preventing the water drainage, thus forming the rough mechanism for foam stabilization. Similarly, for amphiphilic JNPs, they can stabilize foam bubbles by the same mechanism, as depicted in Figure 11. However, unlike soft architectures created by molecular surfactants, the rigidity of the JNPs confer the lamellar structure of the foam created by their self-assembly much more rigidity, and this can also survive foam drainage, leading to the so-called solid-state foams [128]. Pseudo-amphiphilic HNPs were also known to foam [18,129], as well as all kinds of other particles [130], but particularities are expected for foams stabilized by amphiphilic JNPs, especially with respect to the architectures resulting from their templated self-assembly at this water/air interface. In addition, highly performant foaming stabilization ability by JNPs can have practical applications, especially in oil recovery [88,89].

**Figure 12:**
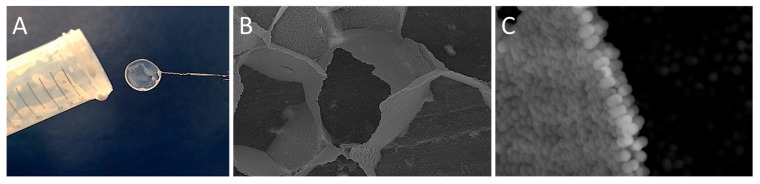
(**A**) Photograph of the foam generated by the functionalized amphiphilic PS/PTSPN JNPs; (**B**) SEM image of the solid foam after drying of the bubble and (**C**) the cross-section of the wall formed after water drainage from the foam lamella, showing highly oriented JNPs. Reproduced with permission from [16], Copyright © 2021 Wiley-VCH GmbH.

Recent work by Mihali and Honciuc [16] demonstrated the formation of polymer-based, snowman-type JNPs obtained via seeded emulsion polymerization and phase separation of a poly(3-(triethoxysilyl)propionitrile) (PTSPN) from PS seed nanoparticles. After asymmetric surface modification of the PS/PTSPN JNPs, namely by hydrolysis of –CN functional group that forms the PTSPN lobe and by coupling to ethylenediamene, the JNPs become strongly amphiphilic, and are capable of foam stabilization by adsorbing at the water/air interface [16]. This leads to the generation of foams stabilized by the oriented assembly of JNPs at the lamellae, as seen in Figure 12. Upon drying, these foams transform into stable solid-state materials with intact lamellar structures formed by bi-layered assemblies of JNPs, where each particle is oriented with its hydrophilic lobe immersed in the aqueous phase and its hydrophobic lobe facing the air, as seen in Figure 12. Synthesized PS/PTSPN JNPs, prior to surface modification, do not stabilize foam, underlining the importance of polarity contrast and that of amphiphilicity in foam stabilization. This behavior, once again, mimics that of classical surfactants but on the colloidal scale, supporting the hypothesis that amphiphilicity is scalable.

**Figure 13:**
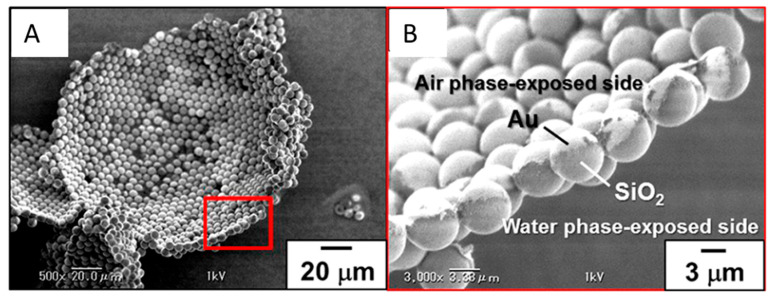
(**A**) Cross-sectional SEM images of bubbles stabilized by the PSg-Au-SiO_2_ Janus particles. (**B**) Zoomed region of the portion indicated, showing oriented JNPs. Reproduced with permission from [15], Copyright © 2019, American Chemical Society.

Fujii et al. [15] demonstrated that polystyrene-grafted Au-SiO_2_ Janus nanoparticles (PS-g-Au-SiO_2_) (~5.8 nm) exhibited monolayer adsorption at the air−water interface and could stabilize bubbles, preventing their coalescence for more than 1 month. In particular, the hydrophobic interactions between the Au sides of the Janus particles drove aggregate formation in water. The JNPs lobes were oriented such that the Au and SiO_2_ lobes were oriented toward the air and water phases, respectively. The hydrophilic SiO_2_ HNPs did not foam, underlining once again the importance of amphiphilicity in interfacial adsorption and foam stabilization. The produced bubbles had the gold-colored appearance, and the sizes ranged from 60 to 700 μm. SEM investigations of the dried foam stabilized by the PS-g-Au-SiO_2_ JNPs clearly demonstrated that the particles are adsorbed as a monolayer at the air−water interface, with the Au and SiO_2_ sides oriented toward the air and water phases, respectively, as seen in Figure 13.

Wang et al. [131] recently reported the preparation of amphiphilic, solid-state JNPs by asymmetric surface modification of silica particles. The synthetic strategy involved site-specific functionalization: one hemisphere was grafted with perfluorodecyltriethoxysilane (PFTS) to impart hydrophobicity, while the opposite hemisphere was either left as unfunctionalized SiO_2_-OH or functionalized with the hydrophilic groups (3-aminopropyl)triethoxysilane (APTES), SiO_2_-NH_2_. This anisotropic surface chemistry rendered the JNPs amphiphilic. The amphiphilic JNPs could effectively stabilize foam structures in aqueous solutions, generating persistent foams.

#### 4.4.3. Templated Self-Assembly at Liquid/Liquid Interfaces: Pickering Emulsions

Emulsions are generated by dispersing one immiscible phase into another. In this process, the surface area of the immiscible system increases significantly the interfacial area and interfacial energy and thus the total energy of the system increases. As a result, the emulsion will collapse by phase separation, unless interfacially active agents—such as surfactants and amphiphiles—are used to minimize the interfacial energy. Emulsions are today widespread in a variety of products and industries. Similarly, Pickering emulsions are a special class of emulsions stabilized by nanoparticles. It has been known that only some particles are able to stabilize emulsions, preferably those that are partially hydrophobic, meaning partially wetted by water, but not as hydrophobic as to completely aggregate [13,132]. Pickering emulsions are interesting for their high stability and coarser dispersion than the classic emulsions stabilized by molecular surfactants. Recently, it has been demonstrated that Pickering emulsions are stabilized well by JNPs and the theoretical grounds were elucidated by Aveyard [133]. Pickering emulsions may benefit from their directional wetting and strong interfacial anchoring of JNPs, leading to greater stability than HNPs. Furthermore, as discussed earlier, the amphiphilic balance of the JNPs determines their preferred position and immersion depth at the oil–water interface [13,134], thus being able to generate W/O or O/W emulsions. These structures not only stabilize emulsions against coalescence but also facilitate templating of hierarchical microspheres via emulsion polymerization techniques.

Jiang and Granick [73] developed a strategy to control the Janus balance of amphiphilic polymer JNPs by tuning their hemispherical volume ratio during Pickering emulsion polymerization. They showed that this balance dictates the particle’s orientation at oil/water interfaces and determines emulsion type and stability. Importantly, particles with balanced amphiphilicity exhibited strong interfacial trapping and promoted self-assembly into ordered monolayers, underscoring the link between amphiphilicity, geometry, and interfacial organization.

**Figure 14:**
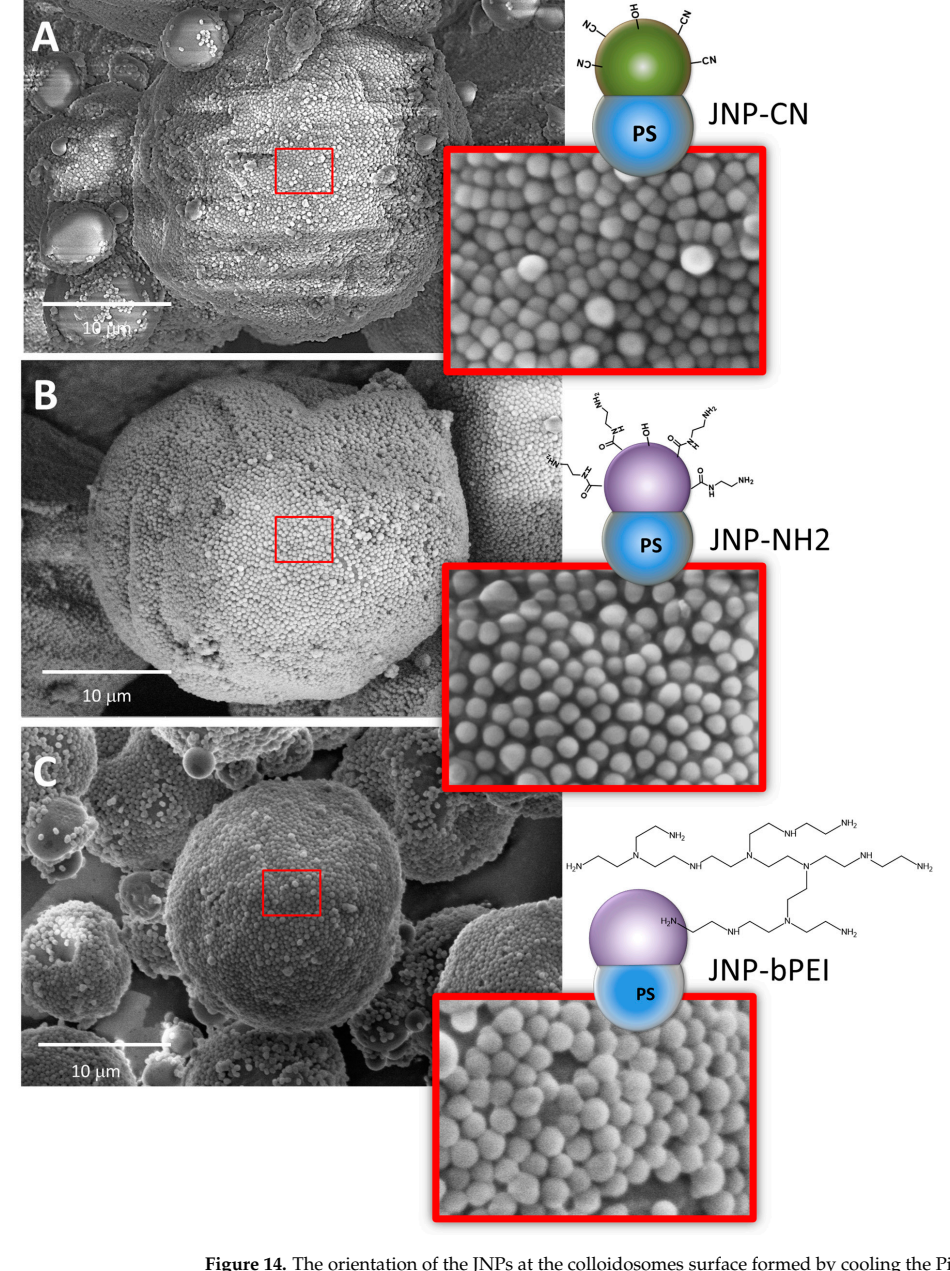
The orientation of the JNPs at the colloidosomes surface formed by cooling the Pickering emulsion of molten wax in water: (**A**) JNP-CN, (**B**) JNPs-NH_2_, and (**C**) JNP-bPEI. Magnified SEM image, on the right side, shows the monolayer of JNPs at the surface of the paraffin wax colloidosome, where (**A**) the JNPs-CN are not oriented, while (**B**) JNPs-NH_2_ and (**C**) JNP-bPEI appear to have a preferred orientation, with the more hydrophilic -NH_2_ and bPEI lobe (light lobe) being oriented towards water and the more hydrophobic PS lobe (dark lobe) towards paraffin wax [16,41]. The red rectangles indicate the location from which the corresponding inset images were taken.

Honciuc and co-workers [3,16,41] demonstrated that the amphiphilicity of JNPs is a pre-requisite for self-assembly into highly oriented monolayers by producing O/W Pickering emulsions from molten wax and water, before allowing the emulsion to cool, thus trapping the JNPs at the interface. For example, by producing polymeric JNPs consisting of a hydrophobic PS lobe and the other lobe functionalized with –CN groups (JNP-CN), which were only weakly amphiphilic but became increasingly hydrophilic after –CN hydrolysis into –COOH groups and strongly hydrophilic upon coupling with ethylenediamine, resulting in JNP-NH_2_ [16], or with branched polyethylene imine, resulting in JNP-bPEI [41], as seen in Figure 14, the authors demonstrated that although the weakly amphiphilic JNP-CN (as demonstrated by their interfacial activity) are capable of self-assembly into a monolayer at molten wax/water interface, they have no preferred orientation at the interface. On the other hand, the JNP-NH_2_ have a strong amphiphilic orientation such that the hydrophobic PS lobe is not visible in the SEM images and is completely immersed in wax, while the more hydrophilic -NH_2_ and bPEI lobes remain oriented toward the water phase.

In another work, Passas-Lagos and Schüth [5] have synthesized hybrid PS/SiO_2_ JNPs and by fine-tuning the relative size ratio of the lobes, they were able to tune the Pickering emulsion phase. Similar works of emulsion phase inversion have been performed by Wu et al. [3] in polymeric PS/P(3-TSPM) JNPs, which also show self-assembly at the wax/water interface and strong preferential orientation of the JNPs monolayers. Similarly, Xie et al. [76] showed the importance of amphiphilic balance in the stabilization and generation of Pickering emulsions.

**Figure 15:**
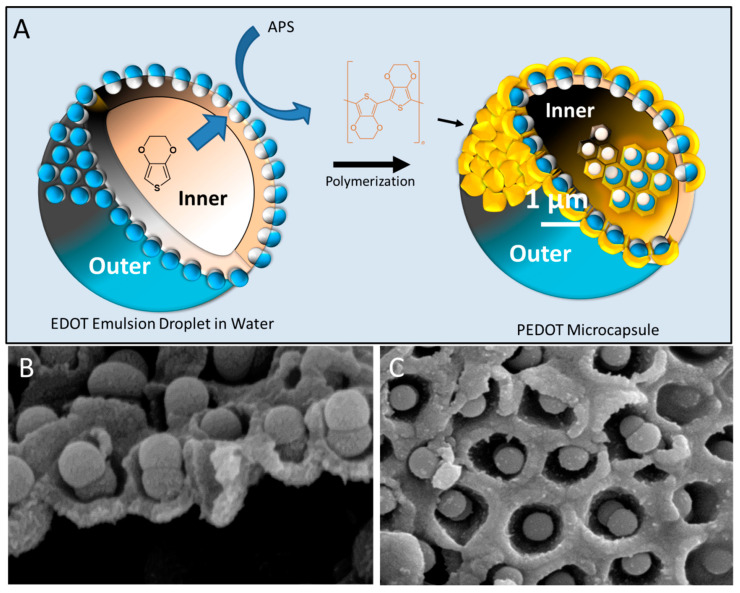
Interfacial polymerization guided by Janus nanoparticle (JNP) monolayers at the surface of EDOT emulsion droplets. (**A**) Schematic representation of the process: Amphiphilic snowman-type JNPs self-assemble at the EDOT/water interface, forming a semi-permeable, monolayer membrane. The aqueous-phase oxidant (APS) diffuses to the interface, where it initiates oxidative polymerization of EDOT. The resulting PEDOT forms at the interface and grows outward into the aqueous phase, templated by the interfacial JNP arrangement. (**B**,**C**) SEM images of the resulting PEDOT microcapsules, showing highly ordered, porous, honeycomb-like architectures. These structures originate from the directional growth of PEDOT through the self-assembled JNP membranes, leading to self-organization. Reproduced with permission from [10], Copyright© 2019 American Chemical Society.

Interestingly, upon adsorption of the JNPs at the interface, the particle monolayer not only provides steric stabilization for the emulsion droplets, preventing their collapse, but they can also serve as membranes to separate, for example, two reaction compartments. For example, Mihali and Honciuc reported [10] that amphiphilic snowman-type JNPs, during the emulsification of ethylenedioxythiophene (EDOT) in water, self-assemble at the oil–water interface into stable monolayers that act as semi-permeable membranes. These interfacial monolayers enable spatial segregation of chemical species, effectively creating two reaction compartments, as seen in Figure 15. In their study, the organic phase containing EDOT monomer is polymerized, while the aqueous phase containing ammonium persulfate (APS) becomes the oxidant initiator. Because the EDOT is emulsified by JNPs, polymerization occurred only at the interface where APS could access the monomer. The JNP membrane played a dual role: stabilizing the emulsion and acting as a selective barrier that controls the location and extent of polymerization. As PEDOT formed at the interface, and because the JNPs were oriented at the interface, the gradual solidification led to self-guided growth of PEDOT into the aqueous phase, resulting in hierarchically organized, honeycomb-like structures as seen in Figure 15. This mimics the compartmentalized growth seen in chemical gardens and highlights how self-assembled JNP monolayers can serve as tunable templates for reaction confinement, spatial control, and structure formation in soft materials chemistry.

#### 4.4.4. Self-Assembly of JNPs in Langmuir–Blodgett Monolayers

The Langmuir–Blodgett (LB) technique is a method for assembling and transferring monolayers of molecules or nanoparticles onto solid substrates. It involves spreading amphiphilic materials at the air–water interface, compressing them into a dense film using movable barriers, and then vertically lifting a solid substrate through the interface to deposit the monolayer [135]. This approach allows precise control over film thickness, particle orientation, and surface coverage, making it valuable for fabricating structured nanomaterials, sensors, and functional coatings [136,137,138].

Upon spreading at the air/water interface from the appropriate solvent, upon solvent evaporation, the amphiphilic JNPs can be compressed into Langmuir monolayers, where they exhibit in-plane ordering and phase transitions reminiscent of surfactant monolayers. The resulting films can be transferred to solid substrates, providing a route to fabricate organized 2D arrays with potential applications in coatings, optics, and sensors. The amphiphilic contrast and particle aspect ratio critically influence packing behavior and the stability of these monolayers.

Sashuk et al. [96] reported the assembly of Au and Ag metal, Janus-type nanoparticles (6–8 nm) at the water/air interface utilizing the Langmuir–Blodgett technique. The JNPs capped with hydrophilic and hydrophobic agents were spread on the surface of water from a CH_2_Cl_2_/MeOH (5:1 *v*/*v*) solvent mixture and were compressed into a hexagonally close-packed monolayer. The pressure–area (p-A) LB isotherm indicated a sharp transition from gas to liquid phase, up to a pressure of 40 mN/m, which corresponded to a well-packed and oriented monolayer of JNPs that could be transferred onto solid substrates by upstroke.

Lenis et al. [139] have also reported the comparative behavior of PS HNPs vs. PS/Au JNPs (~2.4 nm) in Langmuir–Blodgett films, namely that they have studied their collapse behavior at high pressures. Their findings indicate that the PS HNPs films tend to buckle and wrinkle to dissipate the tension, while the JNPs’ LB films, due to their amphiphilicity, tend to collapse by forming subdued domains, resembling the motion of the tectonic plates.

Cheng et al. [140] have constructed a free-standing, 2D gold nanoparticle monolayer film at the water/air interface with asymmetric gold nanoparticle of varying sizes of 25, 50, or 75 nm, which could be grown into an asymmetric 2D Janus gold nanoparticle film.

**Figure 16:**
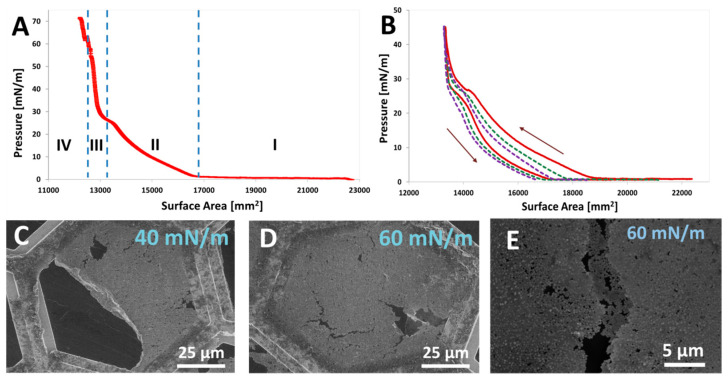
Langmuir–Blodgett isotherm at 25 °C of JNPs-NH_2_ on water subphase: (**A**) Full compression isotherm, where the gas (I), liquid expanded (II), liquid (III) and solid phases (IV) of the particle monolayer are delimited by the dashed lines; (**B**) hysteresis 3 cycles of compression to 45 mN/m, and 3 cycles of expansion to 0 mN/m. SEM images of a freestanding membrane of JNPs-NH_2_ obtained by a Langmuir–Blodgett deposition on copper grids at different surface pressures: (**C**) 40 mN/m; (**D**) 60 mN/m; (**E**) arrangement of JNPs in the monolayers. Reproduced with permission from [16], Copyright © 2021 Wiley-VCH GmbH.

Recent work by Mihali and Honciuc [16] demonstrated the successful formation of freestanding membranes using LB assembly of strongly amphiphilic JNPs with −NH_2_-functionalized lobes (JNPs-NH_2_) synthesized via seeded emulsion polymerization and asymmetric surface modification. These snowman-shaped JNPs, composed of a hydrophobic polystyrene lobe and a hydrophilic amine-terminated siloxane lobe, spontaneously adsorbed at the air–water interface and formed stable, compressible Langmuir monolayers. Upon compression, the LB isotherms revealed distinct phase transitions from a gaseous to a solid phase, with strong lateral interactions driving the formation of a cohesive film, as seen in Figure 16A,B. At high surface pressures (≥60 mN/m), monolayers could be transferred onto solid supports or TEM grids, as shown in Figure 16C–E, yielding freestanding nanoparticle membranes. SEM imaging confirmed the close-packed, mostly oriented arrangement of the JNPs within the monolayers, where the PS lobe pointed toward the air and the NH_2_ lobe faced the water. These LB films have shown a good structural integrity during mechanical manipulation.

To conclude this subsection, the JNPs have played an important role as model systems for understanding the scalability of interfacial phenomena beyond the molecular scale. While the underlying principles of self-assembly are understood in theory, more experimental data from a broader range of systems is still needed to consolidate this understanding. Currently, only a limited number of JNP systems have been shown to reliably form well-defined supra-structures. This continues to drive interest in identifying new JNP platforms capable of stable self-assembly. The architectures of the resulting supra-structures are strongly influenced by the geometric and interfacial characteristics of the JNPs’ building blocks. Each new system must be independently stabilized, characterized, and evaluated for potential applications, which may range from responsive or environmentally aware structures to drug delivery carriers and novel functional materials.

## 5. Conclusions

This review shows that amphiphilicity is a scalable property, extending from molecular surfactants to nanoscale. Experimental data, theoretical models, and simulations confirm that JNPs behave as amphiphilic agents; they lower surface and interfacial tension, spontaneously adsorb at interfaces, and stabilize emulsions and foams. JNPs also self-assemble into supra-structures, both in homogeneous media and at interfaces.

These cumulative behaviors have been demonstrated across various JNP geometries. Their directional interactions, given by their asymmetry, allow orientation at liquid/liquid or liquid/air interfaces, similar to classical surfactants. In Pickering emulsions, Langmuir–Blodgett films, and foam lamellae, JNPs form stable interfacial assemblies and preserve their integrity after their removal from the interface, which makes them valuable for constructing new low-dimensional materials via self-assembly.

Self-assembly of JNPs in homogeneous media also leads to vesicles, micelles, and colloidal capsules, depending on shape and surface functionalization. These results further support the idea that amphiphilicity is not limited to the molecular scale but can be extended to the nanoscale. At the microscale, however, the dominance of gravitational, viscous, and capillary forces may overcome the magnitude of surface and interfacial forces, limiting the observable amphiphilic behavior. Still, JNPs and Janus microparticles are effective model systems to study the scalability of amphiphilic interactions. Their resemblance to surfactants, asymmetric surface engineering, and structural tunability make them valuable in materials design, encapsulation, and interface-driven assembly.

## Figures and Tables

**Table 1:** Aspect ratio and HLB values in the homologous series of JNPs.

* Mass Ratio Between the Lobes in (CPSAA/PS) JNPs	Area PS Lobe (×10^3^ nm^2^)	Area CPSAA Lobe (×10^3^ nm^2^)	A_PS_/A_CPSAA_	^a^ HLB	^b^ *F*_1_	^b^ *F*_2_	^c^ HLB (Weighted)
0	0	70.65	0	20	1	1	20
0.5	4.04	107.87	0.0314	19.3	1	1	19.3
1	14.02	143	0.0981	18.2	1	1	18.2
3	44.26	247.04	0.1791	16.9	1	1	16.9
5	108.37	232.31	0.4665	13.6	1	1	13.6
7	125.6	213.97	0.5871	12.5	1	1	12.5
10	138.7	197.83	0.6984	11.7	1	1	11.7
** JNP in the order of increasing of the P(3-TSPM) polar lobe	^a^ Area PS lobe (×10^3^ nm^2^)	^a^ Area P(3-TSPM) lobe (×10^3^ nm^2^)	JNPs aspect ratio P(3-TSPM)/PS	^b^ HLB	^b^ *F*_1_	^b^ *F*_2_	^c^ HLB (weighted)
PS/P(0.5 mL 3-TSPM)	44.6	15.5	0.35	5	1	1	5
PS/P(1 mL 3-TSPM)	41.5	18.5	0.45	6	1	1	6
PS/P(2 mL 3-TSPM)	40.4	54.1	1.34	11	1	1	11
PS/P(3 mL 3-TSPM)	38.7	60.4	1.56	12	1	1	12
PS/P(4 mL 3-TSPM)	32.6	119.4	3.66	16	1	1	16
*** JNPs in the order of increasing of the P(3-TSPM) polar lobe	^a^ Area PPy-Lobe (×10^3^ nm^2^)	^a^ Area P(3-TSPM) Lobe (×10^3^ nm^2^)	Aspect ratio P(3-TSPM)/PPy	^a^ HLB	^b^ *F*_1_	^b^ *F*_2_	^c^ HLB (weighted)
PPy/P(1 mL 3-TSPM)	177.6	83.2	0.5	6	0.93	0.92	6
PPy/P(2 mL 3-TSPM)	211.8	172.9	0.8	9	0.93	0.92	9
PPy/P(3 mL 3-TSPM)	164.1	318.9	1.9	13	0.93	0.92	13
PPy/P(4 mL 3-TSPM)	152.7	348.7	2.3	14	0.93	0.92	14

Data from * [76], ** [75], and *** [1]. ^a^ Values calculated with Equation (8), assuming *F*_1_ = *F*_2_ = 1. ^b^ *F*_1_ and *F*_2_ were calculated with Equation (9). ^c^ Values calculated with Equation (8).

**Table 2:** Interfacial activity measured by the reduction in interfacial tension (IFT) by JNPs at various water/air and liquid/liquid interfaces.

Janus Nanoparticle Type	Interface	Concentration	Interfacial Tension (IFT) Reduction	Reference
Silica JNPs obtained by functionalization of hemispheres	Oil/water	0.05 wt%	Reduction of IFT to 2.28 mN/m	[84]
Silica JNPs obtained by functionalization of hemispheres with oleic acid	Water/air		ΔIFT = 25 mN/m, greater than bare SiO_2_	[88]
Silica JNPs obtained by functionalization of hemispheres with HMDS/APTS	Water/air		IFT reduced to 58–59 mN/m	[89]
Silica Janus nanoparticles	Paraffin oil/water		IFT reduced from 31.3 to 10.17 mN/m	[30]
Silica JNPs obtained by functionalization of hemispheres with octyl and amino groups	Water/air	0.05 wt%	IFT reduced by JNPs to 36.4 mN/m, greater than that of silica HNPs of 69.8 mN/m	[90]
Silica JNPs obtained by functionalization of hemispheres with octyl and amino groups	Paraffin oil/water	0.05 wt%	IFT reduced by JNPs to 0.067 mN/m, greater than that of silica HNPs of 28.4 mN/m	[90]
Solid polymer PS/PtBA JNPs later hydrolyzed to JNP–COOH by cleaving the tBA groups.	Toluene/water	10 mg/mL	27.9 mN/m	[91]
Solid polymer JNPs, PS− PDIPAEMA/P(3-TSPM) JNPs	Toluene/water		ΔIFT = 11.7 mN/m by JNPs, vs. ΔIFT = 4.0 for the PS− PDIPAEMA HNPs with the same composition as one of the JNP lobes; P(3-TSPM) HNPs not interfacially active	[92]
Solid polymer JNPs, PS− PDIPAEMA/P(3-TSPM) JNPs	Heptane/water		ΔIFT = 15.3 mN/m by JNPs, vs. ΔIFT = 9.1 mN/m for the PS− PDIPAEMA HNPs with the same composition as one of the JNP lobes; P(3-TSPM) HNPs not interfacially active	[92]
Solid polymer JNPs, PS− PDIPAEMA/P(3-TSPM) JNPs	Water/air		ΔIFT = 3.8 mN/m by JNPs, vs. 2.7 mN/m for the PS− PDIPAEMA HNPs with the same composition as one of the JNP lobes; P(3-TSPM) HNPs not interfacially active	[92]
Solid polymer JNPs, obtained by seeded emulsion polymerization PS/P(3-TSPM)+P(3-TESPN) JNPs	Water/air		Variable reduction in the water surface tension with increase in the lobe size of the snowman-type JNPs	[16]
Soft polymer JNPs, made by crosslinking polystyreneblock-polybutadiene-block-poly(methyl methacrylate) (PS-PB-PMMA)	Toluene/water	1 mg/mL	Reduction in the IFT from 34 mN/m to ~19 mN/m.	[93]
Soft polymer JNPs from polystyrene-block-polybutadiene-block-poly(methyl methacrylate)	Toluene/water	1 mg/mL	Reduction in the IFT as a function of the nanoparticle shape to 14 mN/m for cylinders, to 17.5 mN for spheres, to 19 mN/m for disks	[55]
Soft JNPs obtained by selective cross-linking of the polyisoprene domain in the triblock copolymer polystyrene-b-polyisoprene-b-poly(tert-butyl methacrylate) (PS-PI-PtBMA) triblock terpolymers	Water/air	10 mg/mL	Reduction in the IFT from 72 to 63 mN/m	[94]
Soft JNPs obtained by selective cross-linking of the polyisoprene domain in the triblock copolymer polystyrene-b-polyisoprene-b-poly(tert-butyl methacrylate) (PS-PI-PtBMA) triblock terpolymers			Reduction in IFT depends on the HLB value; the largest IFT reduction is for JNPs with lowest HLB, namely ΔIFT = 26 mN/m for HLB = 6 vs. ΔIFT = 10 mN/m for the JNPs with HLB = 6.5	[94]
Metal oxide Fe_3_O_4_ JNPs, capped with hydrophobic C18, C16, C12 alkyl capped magnetic on one side.	Toluene/water		Reduction in the IFT function of the chain length of the capping layer 26.52 ± 0.13 mN/m, in comparison with 27.02 ± 0.07 mN/m and 30.38 ± 0.49 mN/m	[95]
Metallic Au and Ag JNPs obtained by ligand exchange of dodecylamine and decanoic acid with 11-mercaptoundecanoic acid and 1-undecanthiol	Water/air		Strong reduction in the water/air surface tension observed	[96]
Metallic Ag JNPs with 11mercaptoundecanoic acid and 1-undecanthiol capping agents	Water/air Decane/water		DIFTwater/air = 8 mN/m DIFTdecane/water = 9.1 mN/m The interfacial activity of Ag-JNPs was significantly higher compared to silica and PMMA-HNPs of the same size	[97]
Metallic Au JNPs of 3.5 nm diameter (capped with hydrophobic hexanethiolates on one hemisphere and hydrophilic 2-(2mercapto-ethoxy)ethanol on the other)	Water/air	*n* = 17 × 10^12^/5 μL	IFT decreased from 72 to 59.50 mN/m	[98]
Gold–iron oxide Janus nanoparticles	Hexane/water		Review listing findings on greater reduction in IFT by JNPs than HNPs	[99]
Graphene-based amphiphilic Janus nanosheets	Oil/water		Significant changes in IFT observed	[100]

## References

[1] Mihali V., Honciuc A. (2017). Semiconductive Materials with Tunable Electrical Resistance and Surface Polarity Obtained by Asymmetric Functionalization of Janus Nanoparticles. Adv. Mater. Interfaces.

[2] Mihali V., Honciuc A. (2021). Semiconductor–Insulator (Nano-)Couples with Tunable Properties Obtained from Asymmetric Modification of Janus Nanoparticles. ACS Appl. Mater. Interfaces.

[3] Wu D., Chew J.W., Honciuc A. (2016). Polarity Reversal in Homologous Series of Surfactant-Free Janus Nanoparticles: Toward the Next Generation of Amphiphiles. Langmuir.

[4] Wang Y., Wang Y., Breed D.R., Manoharan V.N., Feng L., Hollingsworth A.D., Weck M., Pine D.J. (2012). Colloids with Valence and Specific Directional Bonding. Nature.

[5] Passas-Lagos E., Schüth F. (2015). Amphiphilic Pickering Emulsifiers Based on Mushroom-Type Janus Particles. Langmuir.

[6] Walther A., André X., Drechsler M., Abetz V., Müller A.H.E. (2007). Janus Discs. J. Am. Chem. Soc..

[7] Binks B.P., Fletcher P.D.I. (2001). Particles Adsorbed at the Oil−Water Interface: A Theoretical Comparison between Spheres of Uniform Wettability and “Janus” Particles. Langmuir.

[8] Honciuc A., Toschi F., Sega M. (2019). Amphiphilic Janus Particles at Interfaces. Flowing Matter.

[9] Honciuc A., Solonaru A.-M., Honciuc M. (2023). Pickering Emulsion Polymerization Technology─Toward Nanostructured Materials for Applications in Metal Ion Extractions from Wastewaters. ACS Appl. Polym. Mater..

[10] Mihali V., Honciuc A. (2019). Evolution of Self-Organized Microcapsules with Variable Conductivities from Self-Assembled Nanoparticles at Interfaces. ACS Nano.

[11] Li Y., Liu F., Demirci S., Dey U.K., Rawah T., Chaudary A., Ortega R., Yang Z., Pirhadi E., Huang B. (2025). Two Sides of the Coin: Synthesis and Applications of Janus Particles. Nanoscale.

[12] Honciuc A., Solonaru A.-M., Honciuc M. (2023). Water-Floating Hydrogel Polymer Microsphere Composites for Application in Hydrological Mining of Cu(II) Ions. Nanomaterials.

[13] Honciuc A., Negru O.-I. (2022). Role of Surface Energy of Nanoparticle Stabilizers in the Synthesis of Microspheres via Pickering Emulsion Polymerization. Nanomaterials.

[14] Fujii S., Nakamura Y. (2017). Stimuli-Responsive Bubbles and Foams Stabilized with Solid Particles. Langmuir.

[15] Fujii S., Yokoyama Y., Nakayama S., Ito M., Yusa S., Nakamura Y. (2017). Gas Bubbles Stabilized by Janus Particles with Varying Hydrophilic–Hydrophobic Surface Characteristics. Langmuir.

[16] Mihali V., Honciuc A. (2021). Self-Assembly of Strongly Amphiphilic Janus Nanoparticles into Freestanding Membranes. Adv. Mater. Interfaces.

[17] Kang C., Honciuc A. (2018). Self-Assembly of Janus Nanoparticles into Transformable Suprastructures. J. Phys. Chem. Lett..

[18] Wu D., Mihali V., Honciuc A. (2019). pH-Responsive Pickering Foams Generated by Surfactant-Free Soft Hydrogel Particles. Langmuir.

[19] Wu D., Honciuc A. (2018). Contrasting Mechanisms of Spontaneous Adsorption at Liquid–Liquid Interfaces of Nanoparticles Constituted of and Grafted with pH-Responsive Polymers. Langmuir.

[20] Cheng H., Ma B., Ji A., Yao H., Chen P., Zhai W., Gao S., Shi L., Hu H. (2025). Janus-Structured Micro/Nanomotors: Self-Propelled Mechanisms and Biomedical Applications. Biomater. Res..

[21] Asandulesa M., Solonaru A.-M., Honciuc A. (2024). Effect of Asymmetric Lobe Size on the Molecular Dynamics, Glass Transition, and Dielectric Behavior in Janus Nanoparticles. ACS Appl. Nano Mater..

[22] Mihali V., Jasko P., Skowicki M., Palivan C.G. (2024). Controlled Enzymatic Reactions by Programmed Confinement in Clusters of Polymersomes and Janus Nanoparticles. Mater. Today.

[23] Chen C., Xie Y., Zhang Q., Cao J., Lin J., Tong X., Wu Y., Zhu L., Gao P., Ma J. (2025). Amphiphilic Janus Nanoparticles for a Superhydrophobic Coating on the Enamel Surface to Prevent Caries. Mater. Today Bio.

[24] Chen X., Li W., Fan Q., Liu X., Zhai X., Shi X., Li W., Hong W. (2024). Amphiphilic Janus Nanoparticles for Effective Treatment of Bacterial Pneumonia by Attenuating Inflammation and Targeted Bactericidal Capability. IJN.

[25] Hao M., Chen Y., Leisen J., Whitworth T.J., Xia Y. (2025). Multifunctional Janus Nanoparticles Capable of Anchoring to the Cell Membrane and Serving as “Cellular Backpacks” for Advanced Theranostics. J. Am. Chem. Soc..

[26] He H., Zeng Y., Ma Y., Li K., Liu H., Yang B., Kan Q., Kang G. (2025). Janus Nanoparticles Filled Elastomer Coating for the Improvement of the Low Velocity Impact Performance of Bio-Inspired Composite. Compos. Sci. Technol..

[27] Li Y., Zhang Z., Lv Y., Pei Y., Ding L., Zhang Z., Hu N. (2025). Amphiphilic Janus Nanoparticles for Effective Foam Fractionation of Perfluorooctanoic Acid (PFOA): Effect of Modifier Type. Environ. Res..

[28] Ruan P., He Y., Yang Q., Luo D. (2025). Backscattering Properties of Trigonal Ge-Au Janus Nanoparticles with Different Ambient Refractive Indices in the Visible Solar Spectrum. Optik.

[29] Turpin G., Nguyen D., Sypkes K.I., Vega-Sánchez C., Davey T., Hawkett B.S., Neto C. (2025). Encapsulation of Oil Droplets Using Film-Forming Janus Nanoparticles. Langmuir.

[30] Xu K., Yang Z., Li X., Jiang B., Shen H., Lin M., Dong Z. (2024). Hydrophilic–Lipophilic Regulation by Amphiphilic Silicon-Based Janus Nanoparticles. ACS Appl. Nano Mater..

[31] Ma Y., Yu J., Sun M., Chen B., Zhou X., Ye C., Guan Z., Guo W., Wang G., Lu S. (2022). Confined Growth of Silver–Copper Janus Nanostructures with {100} Facets for Highly Selective Tandem Electrocatalytic Carbon Dioxide Reduction. Adv. Mater..

[32] Zhao H., Xing Y., Hao W., Fan B., Zhang R. (2025). Gold–Silica Janus Nanoparticles for Multimodal Therapy against Tumors in Vitro. ChemistrySelect.

[33] Wu H., Luo Y., Li G., Yuan Y., Chang J., Kang N., Hou J. (2025). Enhanced Oil Recovery Using Amphiphilic Nanomaterials with Tailored Functionalities: A Review. J. Mol. Liq..

[34] Tohidi Z., Teimouri A., Jafari A., Gharibshahi R., Omidkhah M.R. (2022). Application of Janus Nanoparticles in Enhanced Oil Recovery Processes: Current Status and Future Opportunities. J. Pet. Sci. Eng..

[35] Rahiminezhad Z., Tamaddon A.M., Borandeh S., Abolmaali S.S. (2020). Janus Nanoparticles: New Generation of Multifunctional Nanocarriers in Drug Delivery, Bioimaging and Theranostics. Appl. Mater. Today.

[36] Rabanel J.-M., Adibnia V., Tehrani S.F., Sanche S., Hildgen P., Banquy X., Ramassamy C. (2019). Nanoparticle Heterogeneity: An Emerging Structural Parameter Influencing Particle Fate in Biological Media?. Nanoscale.

[37] Zhang X., Fu Q., Duan H., Song J., Yang H. (2021). Janus Nanoparticles: From Fabrication to (Bio)Applications. ACS Nano.

[38] Su H., Hurd Price C.-A., Jing L., Tian Q., Liu J., Qian K. (2019). Janus Particles: Design, Preparation, and Biomedical Applications. Mater. Today Bio.

[39] Liu Y., Chen L., Chen Z., Liu M., Li X., Kou Y., Hou M., Wang H., Li X., Tian B. (2023). Multifunctional Janus Nanoplatform for Efficiently Synergistic Theranostics of Rheumatoid Arthritis. ACS Nano.

[40] Duan Z., Han J., Liu Y., Zhao X., Wang B., Cao S., Wu D. (2024). A Polymeric ^1^H/^19^F Dual-Modal MRI Contrast Agent with a Snowman-like Janus Nanostructure. J. Mater. Chem. B.

[41] Pauli O., Honciuc A. (2022). Extraction of Metal Ions by Interfacially Active Janus Nanoparticles Supported by Wax Colloidosomes Obtained from Pickering Emulsions. Nanomaterials.

[42] Kirillova A., Marschelke C., Synytska A. (2019). Hybrid Janus Particles: Challenges and Opportunities for the Design of Active Functional Interfaces and Surfaces. ACS Appl. Mater. Interfaces.

[43] Cho I., Lee K.-W. (1985). Morphology of Latex Particles Formed by Poly(Methyl Methacrylate)-Seeded Emulsion Polymerization of Styrene. J. Appl. Polym. Sci..

[44] Chen Y.-C., Dimonie V., El-Aasser M.S. (1991). Interfacial Phenomena Controlling Particle Morphology of Composite Latexes. J. Appl. Polym. Sci..

[45] Chen Y.C., Dimonie V., El-Aasser M.S. (1992). Role of Surfactant in Composite Latex Particle Morphology. J. Appl. Polym. Sci..

[46] Li X., Chen L., Cui D., Jiang W., Han L., Niu N. (2022). Preparation and Application of Janus Nanoparticles: Recent Development and Prospects. Coord. Chem. Rev..

[47] Liu Y., Wang J., Shao Y., Deng R., Zhu J., Yang Z. (2022). Recent Advances in Scalable Synthesis and Performance of Janus Polymer/Inorganic Nanocomposites. Prog. Mater. Sci..

[48] Casagrande C., Fabre P., Raphael E., Veyssie M. (1989). Janus Beads—Realization and Behavior at Water Oil Interfaces. Europhys. Lett..

[49] Casagrande C., Veyssie M. (1988). «Grains Janus»: Réalisation et premières observations des propriétés interfaciales; Janus Beads: Realization and first observation of interfacial properties. Proc. Acad. Ser. II.

[50] Erhardt R., Böker A., Zettl H., Kaya H., Pyckhout-Hintzen W., Krausch G., Abetz V., Müller A.H.E. (2001). Janus Micelles. Macromolecules.

[51] Erhardt R., Zhang M., Böker A., Zettl H., Abetz C., Frederik P., Krausch G., Abetz V., Müller A.H.E. (2003). Amphiphilic Janus Micelles with Polystyrene and Poly(Methacrylic Acid) Hemispheres. J. Am. Chem. Soc..

[52] Gröschel A.H., Walther A., Löbling T.I., Schmelz J., Hanisch A., Schmalz H., Müller A.H.E. (2012). Facile, Solution-Based Synthesis of Soft, Nanoscale Janus Particles with Tunable Janus Balance. J. Am. Chem. Soc..

[53] Gröschel A.H., Walther A., Löbling T.I., Schacher F.H., Schmalz H., Müller A.H.E. (2013). Guided Hierarchical Co-Assembly of Soft Patchy Nanoparticles. Nature.

[54] Walther A., Hoffmann M., Müller A.H.E. (2008). Emulsion Polymerization Using Janus Particles as Stabilizers. Angew. Chem. Int. Ed..

[55] Ruhland T.M., Gröschel A.H., Ballard N., Skelhon T.S., Walther A., Müller A.H.E., Bon S.A.F. (2013). Influence of Janus Particle Shape on Their Interfacial Behavior at Liquid–Liquid Interfaces. Langmuir.

[56] Ruhland T.M., Gröschel A.H., Walther A., Müller A.H.E. (2011). Janus Cylinders at Liquid–Liquid Interfaces. Langmuir.

[57] Lattuada M., Hatton T.A. (2011). Synthesis, Properties and Applications of Janus Nanoparticles. Nano Today.

[58] Walther A., Müller A.H.E. (2013). Janus Particles: Synthesis, Self-Assembly, Physical Properties, and Applications. Chem. Rev..

[59] Honciuc A. (2021). Chemistry of Functional Materials Surfaces and Interfaces: Fundamentals and Applications.

[60] Monnard P.-A., Deamer D.W., Luisi P.L., Stano P. (2011). Membrane Self-Assembly Processes: Steps Toward the First Cellular Life. The Minimal Cell.

[61] Israelachvili J.N. (2011). 20-Soft and Biological Structures. Intermolecular and Surface Forces.

[62] Israelachvili J. (1994). Self-Assembly in Two Dimensions: Surface Micelles and Domain Formation in Monolayers. Langmuir.

[63] Wang C., Wang Z., Zhang X. (2012). Amphiphilic Building Blocks for Self-Assembly: From Amphiphiles to Supra-Amphiphiles. Acc. Chem. Res..

[64] Israelachvili J.N. (2011). 17-Adhesion and Wetting Phenomena. Intermolecular and Surface Forces: Revised.

[65] Kang C., Honciuc A. (2018). Influence of Geometries on the Assembly of Snowman-Shaped Janus Nanoparticles. ACS Nano.

[66] Griffin C.W. (1955). Calculation of HLB Values of Non-Ionic Surfactants. J. Soc. Cosmet. Chem..

[67] Pasquali R.C., Taurozzi M.P., Bregni C. (2008). Some Considerations about the Hydrophilic–Lipophilic Balance System. Int. J. Pharm..

[68] Binks B.P. (2002). Particles as Surfactants—Similarities and Differences. Curr. Opin. Colloid Interface Sci..

[69] Pieranski P. (1980). Two-Dimensional Interfacial Colloidal Crystals. Phys. Rev. Lett..

[70] Binks B.P., Lumsdon S.O. (2000). Effects of Oil Type and Aqueous Phase Composition on Oil–Water Mixtures Containing Particles of Intermediate Hydrophobicity. Phys. Chem. Chem. Phys..

[71] Ondarçuhu T., Fabre P., Raphaël E., Veyssié M. (1990). Specific Properties of Amphiphilic Particles at Fluid Interfaces. J. Phys. Fr..

[72] Borówko M., Staszewski T. (2023). Hybrid Nanoparticles at Fluid–Fluid Interfaces: Insight from Theory and Simulation. IJMS.

[73] Jiang S., Granick S. (2008). Controlling the Geometry (Janus Balance) of Amphiphilic Colloidal Particles. Langmuir.

[74] Lee K., Yu Y. (2018). Lipid Bilayer Disruption by Amphiphilic Janus Nanoparticles: The Role of Janus Balance. Langmuir.

[75] Wu D., Binks B.P., Honciuc A. (2018). Modeling the Interfacial Energy of Surfactant-Free Amphiphilic Janus Nanoparticles from Phase Inversion in Pickering Emulsions. Langmuir.

[76] Xie S., Chen S., Zhu Q., Li X., Wang D., Shen S., Jin M., Zhou G., Zhu Y., Shui L. (2020). Janus Nanoparticles with Tunable Amphiphilicity for Stabilizing Pickering-Emulsion Droplets via Assembly Behavior at Oil–Water Interfaces. ACS Appl. Mater. Interfaces.

[77] Rosen M.J., Cohen A.W., Dahanayake M., Hua X.Y. (1982). Relationship of Structure to Properties in Surfactants. 10. Surface and Thermodynamic Properties of 2-Dodecyloxypoly (Ethenoxyethanol) s, C_12_H_25_ (OC_2_H_4_) xOH, in Aqueous Solution. J. Phys. Chem..

[78] Rosen M.J., Li F., Morrall S.W., Versteeg D.J. (2001). The Relationship between the Interfacial Properties of Surfactants and Their Toxicity to Aquatic Organisms. Environ. Sci. Technol..

[79] Milton J.R. (2004). Surfactants and Interfacial, Phenomena.

[80] Inada C., Kobayashi Y., Yamakawa M., Kitagawa A. (2024). Interfacial Assembly and Properties of Amphiphilic Polymer-Grafted Nanoparticles: Effect of Chemical Design and Density of Grafted Polymers. Colloids Surf. A Physicochem. Eng. Asp..

[81] Kralchevsky P.A., Nagayama K. (2000). Capillary Interactions between Particles Bound to Interfaces, Liquid Films and Biomembranes. Adv. Colloid Interface Sci..

[82] Vassileva N.D., van den Ende D., Mugele F., Mellema J. (2005). Capillary Forces between Spherical Particles Floating at a Liquid−Liquid Interface. Langmuir.

[83] Okubo T. (1995). Surface Tension of Structured Colloidal Suspensions of Polystyrene and Silica Spheres at the Air-Water Interface. J. Colloid Interface Sci..

[84] Wu H., Gao K., Lu Y., Meng Z., Gou C., Li Z., Yang M., Qu M., Liu T., Hou J. (2020). Silica-Based Amphiphilic Janus Nanofluid with Improved Interfacial Properties for Enhanced Oil Recovery. Colloids Surf. A.

[85] Giraldo L.J., Gallego J., Villegas J.P., Franco C.A., Cortés F.B. (2019). Enhanced Waterflooding with NiO/SiO2 0-D Janus Nanoparticles at Low Concentration. J. Pet. Sci. Eng..

[86] Cao J., Chen Y., Xu G., Wang X., Li Y., Zhao S., Liu C., Wang X. (2022). Study on Interface Regulation Effects of Janus Nanofluid for Enhanced Oil Recovery. Colloids Surf. A Physicochem. Eng. Asp..

[87] Liu P., Li X., Yu H., Niu L., Yu L., Ni D., Zhang Z. (2020). Functional Janus-SiO2 Nanoparticles Prepared by a Novel “Cut the Gordian Knot” Method and Their Potential Application for Enhanced Oil Recovery. ACS Appl. Mater. Interfaces.

[88] Saeedi Dehaghani A.H., Gharibshahi R., Mohammadi M. (2025). Synthesis and Performance Analysis of Novel SiO_2_ Janus Nanoparticles for Enhancing Gas Foam Injection in Oil Reservoirs. Sci. Rep..

[89] Saeedi Dehaghani A.H., Gharibshahi R., Mohammadi M. (2023). Utilization of Synthesized Silane-Based Silica Janus Nanoparticles to Improve Foam Stability Applicable in Oil Production: Static Study. Sci. Rep..

[90] Tang S., Sun Z., Dong Y., Zhu Y., Hu H., Wang R., Liao H., Dai Q. (2024). Preparation of Amphiphilic Janus-SiO_2_ Nanoparticles and Evaluation of the Oil Displacement Effect. ACS Omega.

[91] McGlasson A., Morgenthaler E., Bradley L.C., Russell T.P. (2024). On the Interfacial Assembly of Anisotropic Amphiphilic Janus Particles. Adv. Funct. Mater..

[92] Wu D., Honciuc A. (2018). Design of Janus Nanoparticles with pH-Triggered Switchable Amphiphilicity for Interfacial Applications. ACS Appl. Nano Mater..

[93] Jiang Y., Löbling T.I., Huang C., Sun Z., Müller A.H.E., Russell T.P. (2017). Interfacial Assembly and Jamming Behavior of Polymeric Janus Particles at Liquid Interfaces. ACS Appl. Mater. Interfaces.

[94] Schröder J.H., Doroshenko M., Pirner D., Mauer M.E.J., Förster B., Boyko V., Reck B., Roschmann K.J., Müller A.H.E., Förster S. (2016). Interfacial Stabilization by Soft Janus Nanoparticles. Polymer.

[95] Yang F., He X., Tan W., Liu G., Yi T., Lu Q., Wei X., Xie H., Long Q., Wang G. (2022). Adhesion-Shielding Based Synthesis of Interfacially Active Magnetic Janus Nanoparticles. J. Colloid Interface Sci..

[96] Sashuk V., Hołyst R., Wojciechowski T., Fiałkowski M. (2012). Close-Packed Monolayers of Charged Janus-Type Nanoparticles at the Air–Water Interface. J. Colloid Interface Sci..

[97] Fernandez-Rodriguez M.A., Ramos J., Isa L., Rodriguez-Valverde M.A., Cabrerizo-Vilchez M.A., Hidalgo-Alvarez R. (2015). Interfacial Activity and Contact Angle of Homogeneous, Functionalized, and Janus Nanoparticles at the Water/Decane Interface. Langmuir.

[98] Fernandez-Rodriguez M.A., Song Y., Rodríguez-Valverde M.Á., Chen S., Cabrerizo-Vilchez M.A., Hidalgo-Alvarez R. (2014). Comparison of the Interfacial Activity between Homogeneous and Janus Gold Nanoparticles by Pendant Drop Tensiometry. Langmuir.

[99] Correia E.L., Brown N., Razavi S. (2021). Janus Particles at Fluid Interfaces: Stability and Interfacial Rheology. Nanomaterials.

[100] Luo D., Wang F., Zhu J., Cao F., Liu Y., Li X., Willson R.C., Yang Z., Chu C.-W., Ren Z. (2016). Nanofluid of Graphene-Based Amphiphilic Janus Nanosheets for Tertiary or Enhanced Oil Recovery: High Performance at Low Concentration. Proc. Natl. Acad. Sci. USA.

[101] Deng K., Luo Z., Tan L., Quan Z. (2020). Self-Assembly of Anisotropic Nanoparticles into Functional Superstructures. Chem. Soc. Rev..

[102] Agrawal G., Agrawal R. (2019). Janus Nanoparticles: Recent Advances in Their Interfacial and Biomedical Applications. ACS Appl. Nano Mater..

[103] Garbuzenko O.B., Winkler J., Tomassone M.S., Minko T. (2014). Biodegradable Janus Nanoparticles for Local Pulmonary Delivery of Hydrophilic and Hydrophobic Molecules to the Lungs. Langmuir.

[104] Li B., Wang M., Chen K., Cheng Z., Chen G., Zhang Z. (2015). Synthesis of Biofunctional Janus Particles. Macromol. Rapid Commun..

[105] Discher D.E., Eisenberg A. (2002). Polymer Vesicles. Science.

[106] Jain S., Bates F.S. (2003). On the Origins of Morphological Complexity in Block Copolymer Surfactants. Science.

[107] Hartgerink J.D., Beniash E., Stupp S.I. (2001). Self-Assembly and Mineralization of Peptide-Amphiphile Nanofibers. Science.

[108] Nagarajan R. (2002). Molecular Packing Parameter and Surfactant Self-Assembly: The Neglected Role of the Surfactant Tail. Langmuir.

[109] Hong L., Cacciuto A., Luijten E., Granick S. (2008). Clusters of Amphiphilic Colloidal Spheres. Langmuir.

[110] Borówko M., Staszewski T., Tomasik J. (2023). Janus Ligand-Tethered Nanoparticles at Liquid–Liquid Interfaces. J. Phys. Chem. B.

[111] Baran Ł., Borówko M., Rżysko W., Smołka J. (2023). Amphiphilic Janus Particles Confined in Symmetrical and Janus-Like Slits. ACS Omega.

[112] Baran Ł., Borówko M., Rżysko W. (2020). Self-Assembly of Amphiphilic Janus Particles Confined between Two Solid Surfaces. J. Phys. Chem. C.

[113] Liu F., Goyal S., Forrester M., Ma T., Miller K., Mansoorieh Y., Henjum J., Zhou L., Cochran E., Jiang S. (2019). Self-Assembly of Janus Dumbbell Nanocrystals and Their Enhanced Surface Plasmon Resonance. Nano Lett..

[114] Hu H., Ji F., Xu Y., Yu J., Liu Q., Chen L., Chen Q., Wen P., Lifshitz Y., Wang Y. (2016). Reversible and Precise Self-Assembly of Janus Metal-Organosilica Nanoparticles through a Linker-Free Approach. ACS Nano.

[115] Kirillova A., Stoychev G., Ionov L., Synytska A. (2014). Self-Assembly Behavior of Hairy Colloidal Particles with Different Architectures: Mixed versus Janus. Langmuir.

[116] Gao W., Pei A., Feng X., Hennessy C., Wang J. (2013). Organized Self-Assembly of Janus Micromotors with Hydrophobic Hemispheres. J. Am. Chem. Soc..

[117] Nie Z., Li W., Seo M., Xu S., Kumacheva E. (2006). Janus and Ternary Particles Generated by Microfluidic Synthesis: Design, Synthesis, and Self-Assembly. J. Am. Chem. Soc..

[118] Li W., Liu Y., Brett G., Gunton J.D. (2012). Encapsulation by Janus Spheroids. Soft Matter.

[119] Liu Y., Li W., Perez T., Gunton J.D., Brett G. (2012). Self Assembly of Janus Ellipsoids. Langmuir.

[120] Li W., Gunton J.D. (2013). Self-Assembly of Janus Ellipsoids II: Janus Prolate Spheroids. Langmuir.

[121] Shin H., Schweizer K.S. (2013). Theory of Two-Dimensional Self-Assembly of Janus Colloids: Crystallization and Orientational Ordering. Soft Matter.

[122] Whitelam S., Bon S.A.F. (2010). Self-Assembly of Amphiphilic Peanut-Shaped Nanoparticles. J. Chem. Phys..

[123] Tsonchev S., Schatz G.C., Ratner M.A. (2003). Hydrophobically-Driven Self-Assembly: A Geometric Packing Analysis. Nano Lett..

[124] Kraft D.J., Ni R., Smallenburg F., Hermes M., Yoon K., Weitz D.A., van Blaaderen A., Groenewold J., Dijkstra M., Kegel W.K. (2012). Surface Roughness Directed Self-Assembly of Patchy Particles into Colloidal Micelles. Proc. Natl. Acad. Sci. USA.

[125] Chen Q., Bae S.C., Granick S. (2011). Directed Self-Assembly of a Colloidal Kagome Lattice. Nature.

[126] Chen Q., Diesel E., Whitmer J.K., Bae S.C., Luijten E., Granick S. (2011). Triblock Colloids for Directed Self-Assembly. J. Am. Chem. Soc..

[127] Kang C., Honciuc A. (2019). Versatile Triblock Janus Nanoparticles: Synthesis and Self-Assembly. Chem. Mater..

[128] Kraynik A.M. (2003). Foam Structure: From Soap Froth to Solid Foams. MRS Bull..

[129] Blute I., Pugh R.J., van de Pas J., Callaghan I. (2009). Industrial Manufactured Silica Nanoparticle Sols. 2: Surface Tension, Particle Concentration, Foam Generation and Stability. Colloids Surf. A: Physicochem. Eng. Asp..

[130] Fameau A.-L., Salonen A. (2014). Effect of Particles and Aggregated Structures on the Foam Stability and Aging. Comptes Rendus. Phys..

[131] Wang K., Wang G., Lu C., Wang Y. (2018). Preparation of Amphiphilic Janus Particles and Their Application in Stabilising Foams. Micro. Nano Lett..

[132] Finkle P., Draper H.D., Hildebrand J.H. (1923). The Theory of Emulsification. J. Am. Chem. Soc..

[133] Aveyard R. (2012). Can Janus Particles Give Thermodynamically Stable Pickering Emulsions?. Soft Matter.

[134] Honciuc A., Negru O.-I. (2021). NanoTraPPED—A New Method for Determining the Surface Energy of Nanoparticles via Pickering Emulsion Polymerization. Nanomaterials.

[135] Ariga K., Yamauchi Y., Mori T., Hill J.P. (2013). 25th Anniversary Article: What Can Be Done with the Langmuir-Blodgett Method? Recent Developments and Its Critical Role in Materials Science. Adv. Mater..

[136] Bardosova M., Pemble M.E., Povey I.M., Tredgold R.H. (2010). The Langmuir-Blodgett Approach to Making Colloidal Photonic Crystals from Silica Spheres. Adv. Mater..

[137] Parchine M., McGrath J., Bardosova M., Pemble M.E. (2016). Large Area 2D and 3D Colloidal Photonic Crystals Fabricated by a Roll-to-Roll Langmuir–Blodgett Method. Langmuir.

[138] Honciuc A., Negru O.-I. (2023). Asymmetrically Nanostructured 2D Janus Films Obtained from Pickering Emulsions Polymerized in a Langmuir–Blodgett Trough. Micromachines.

[139] Lenis J., Razavi S., Cao K.D., Lin B., Lee K.Y.C., Tu R.S., Kretzschmar I. (2015). Mechanical Stability of Polystyrene and Janus Particle Monolayers at the Air/Water Interface. J. Am. Chem. Soc..

[140] Cheng Q., Song L., Lin H., Yang Y., Huang Y., Su F., Chen T. (2020). Free-Standing 2D Janus Gold Nanoparticles Monolayer Film with Tunable Bifacial Morphologies via the Asymmetric Growth at Air–Liquid Interface. Langmuir.

